# Comprehensive self-antigen screening to assess cross-reactivity in promiscuous T-cell receptors

**DOI:** 10.3389/fimmu.2025.1719827

**Published:** 2026-01-05

**Authors:** Govinda Sharma, Fei Teng, James Round, Sophie A. Sneddon, Sarania Sivasothy, Scott D. Brown, Robert A. Holt

**Affiliations:** 1 Michael Smith Genome Sciences Centre, British Columbia Cancer Research Institute, Vancouver, BC, Canada; 2 Immfinity Biotechnologies Inc, Vancouver, BC, Canada; 3 Department of Molecular Biology and Biochemistry, Simon Fraser University, Burnaby, BC, Canada; 4 Department of Medical Genetics, University of British Columbia, Vancouver, BC, Canada

**Keywords:** adaptive immune repertoires, cell therapy, epitope discovery, preclinical development, T cell receptor

## Abstract

T cell receptor (TCR) therapeutics are an emerging modality of biologic and cell-based medicines with the unique ability to target intracellular antigens and finely discriminate between healthy and infected or mutated cells. An obstacle to the development of new TCR therapeutics is the difficulty in engineering these proteins for enhanced therapeutic efficacy while avoiding introduction of unexpected off-target autoreactivity. In this study, we conduct functional high-throughput screening to profile all possible genome-encoded peptides for ability to trigger response in an engineered candidate therapeutic TCR and assess risk of off-target toxicity. We used multiple approaches for constructing comprehensive self-antigen cell libraries and highlight key considerations for designing toxicity screening campaigns using high-throughput TCR profiling in a live cell context. We then use Tope-seq to screen epitope libraries against a model therapeutic candidate TCR and show that this strategy can be used to detect known cross-reactive epitopes from libraries of >5 × 10^5^ unique peptide-coding sequences at a significance threshold of p < 0.01 in first-pass bulk screening. We also incorporate strategies for iterative biopanning and bioinformatic refinement to improve sensitivity and accuracy and demonstrate here the first proof-of-principle for functional TCR screening on a library of >2 × 10^7^ peptide-coding DNA fragments, further advancing the potential of *in vitro* approaches to perform unbiased TCR epitope discovery.

## Introduction

T cells are a critical piece of the vertebrate adaptive immune system responsible for removing pathogens and mutated cells from the body. Central to the function of T cells is the T cell receptor (TCR), a membrane-anchored immunoglobulin family member protein generated by the programmed somatic rearrangement of germline-encoded VDJ genes. T cell populations within individuals are composed of repertoires of millions of clones each expressing a uniquely recombined configuration of TCR gene segments. Each TCR interacts with short peptide epitopes, which are derived from proteolytic turnover of cellular proteins, loaded onto major histocompatibility complex (MHC) molecules in the endoplasmic reticulum and displayed on the exterior surface. The resulting peptide/MHC (pMHC) complexes represent a sampling of the proteomic landscape of individual cells, including proteins expressed by foreign organisms, and are inspected by peripheral T cells. Individual TCR clonotypes have distinct binding preferences with respect to pMHC ligands and, if a positive TCR-pMHC interaction is formed, T cell effector functions are initiated. Collectively, the repertoire of T cells, each harboring their own TCR, provides a broad range of protection from intracellular pathogens or tumorigenesis (1).

The semi-randomized process of TCR generation during T cell development is regulated in the thymus, where nascent TCR proteins are exposed to self-antigen pMHC in a process known as central tolerance (2). Rearranged clonotypes responding strongly to self-antigen in the thymus are deleted, resulting in a peripheral repertoire of TCR tolerized to healthy tissue, yet poised to respond to foreign or altered peptides. In recent years, efforts to leverage TCRs as therapeutic agents (3, 4), particularly in oncology, have been pursued by identifying TCR with demonstrated response against specific peptides from tumor associated antigens, typically from searching through patient or donor tissue, and converting these proteins into either soluble T cell engager biologics or as recombinant TCR-T cell therapies. In these therapeutic contexts, applying allogeneically sourced and engineered TCR in supraphysiological amounts circumvent natural mechanisms of central tolerance and, thus, comprehensive assessment of autoreactivity must be recapitulated in an *ex vivo* context to ensure safety and tolerability in new TCR-based therapeutics.

Profiling autoreactivity in TCR responses has remained a challenge in the field because of the tendency of these responses to be precise – capable of distinguishing between peptide sequences differing by a single amino acid (5) – while simultaneously being highly cross-reactive, capable of responding to a vast range of millions of potential epitopes (6). This interplay between fine discrimination and receptor-ligand promiscuity is governed by a number of biophysical and signaling phenomena such as serial triggering, kinetic proofreading, and steric interactions at the immune synapse (7–9). Methods relying solely on affinity between TCR and pMHC, such as soluble multimeric pMHC staining reagents (10) or pMHC yeast/viral display (11), are often used as a proxy for functional triggering of T cell responses but run the risk of ignoring low affinity peptides with high functional potential (12, 13). Further, animal models, even those with humanized HLA loci, are not adequate for assessing human TCR because of cross-species proteomic mismatches. Machine learning based predictive tools have recently emerged as a major area of innovation towards addressing this challenge, however, the considerable lack of functionally validated training data has meant that these approaches have not yet emerged as a practical solution (14). Performing comprehensive functional assessment of self cross-reactivity early in the therapeutic TCR development pipeline is essential for scaling up the lead identification and pre-clinical characterization of novel TCR-based biologics and cell therapies (15).

**Figure 1:**
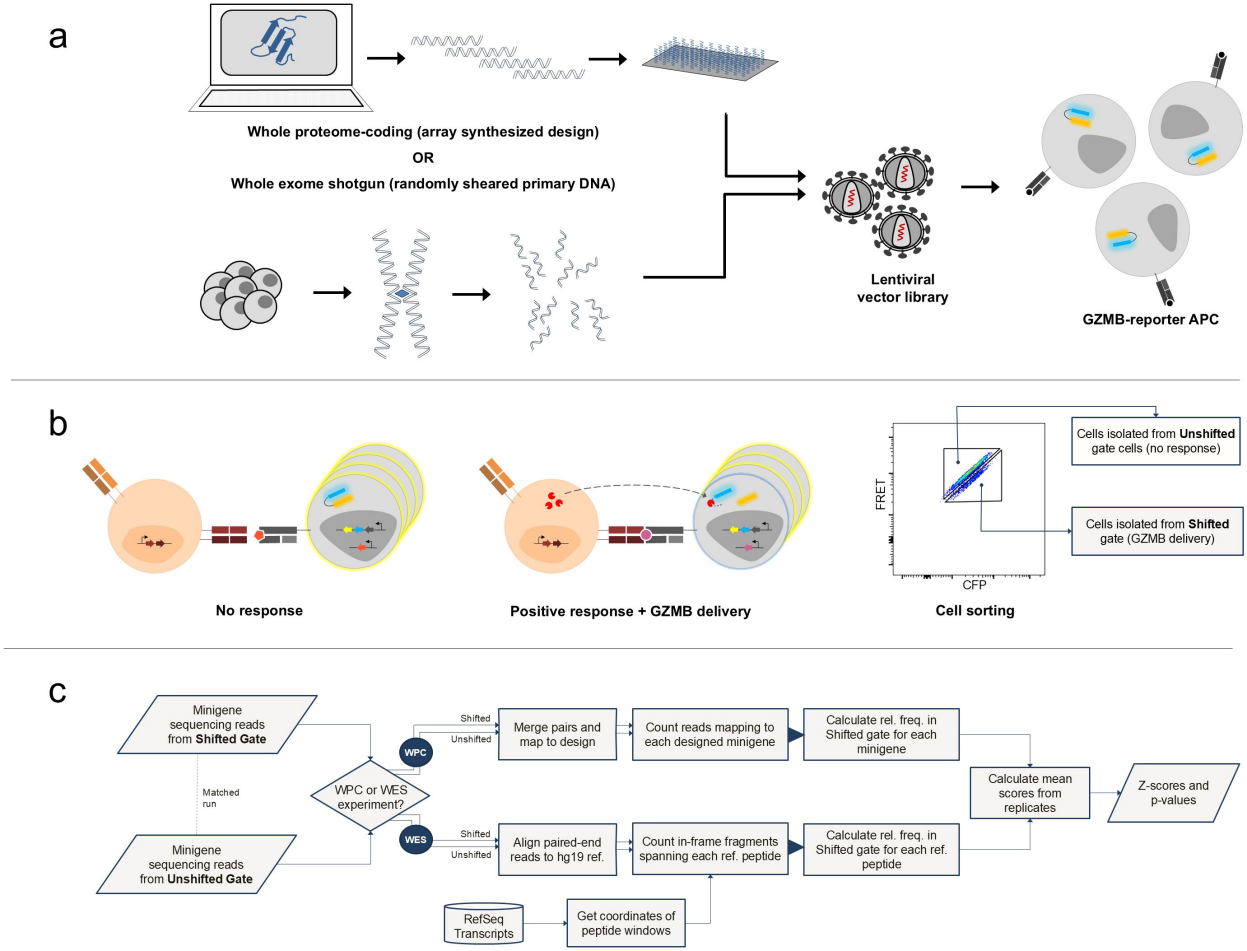
Schematic summary of Tope-seq platform. The parallel approaches for self-antigen minigene library development are illustrated in **(a)**. In one arm, the whole proteome coding (WPC) format library is created from primary amino acid sequences of all proteins identified in the human reference proteome accessed and fragmented in silico using a defined tiling scheme. The extracted amino acid sequences are then converted to DNA sequences by random codon back-translation and individually synthesized as single-stranded oligonucleotides on an array-based platform. After synthesis, DNA fragments are liberated from the array, pooled, and used to produce a lentiviral vector library. In another arm of library construction, a whole exome shotgun (WES) library was generated using primary genomic DNA from live normal human cells isolated and physically fragmented by sonication. The resultant double-stranded DNA fragments are then subjected to exome capture by RNA hybridization probes, end-polished, and adapterized for PCR amplification prior being used to produce lentiviral vector library. Library virus from either stream is used to transduce sAPC in preparation for Tope-seq based screening. The principle of the Tope-seq assay format is represented in **(b)**. Activated effector T cells transduced with the exogenous TCR-of-interest are co-cultured with sAPC harboring an integrated self-antigen minigene library. When TCR-T cells encounter a target cell containing a minigene coding for a cognate epitope, GZMB molecules are delivered to the offending target where they cleave target-encoded ECFP-EYFP fusion proteins separated by a GZMB substrate peptide linker. The resulting loss of FRET signal (or ‘FRET-shift’) upon cleavage is monitored in flow cytometry and the library cell population is isolated by FACS to capture the Shifted cells and the counterpart Unshifted fraction. Recovered cells are characterized by targeted NGS to identify the virally encoded minigenes present in the cells from both matched gates. To quantify minigene enrichment in the Tope-seq experiment, a bioinformatic pipeline outlined in **(c)** is used. For WPC library-based screening, matched Shifted and Unshifted gate minigene sequencing reads are paired-end merged, normalized to sample total read depth, and aligned to designed library reference sequences. Once processed, enrichment scores for each individual minigene in the library are calculated by determining the relative frequency of normalized counts in the Shifted gate (rf_s_) as a proportion of the total across both gates. For WES libraries, processing is performed by mapping paired-end reads to the reference human genome and using a sliding reference peptide window to scan all k-mer size ranges (from 8 to 11 residues in length) across the coding genome. In-frame minigenes traversing each window are counted and normalized to read depth for each matched gate dataset prior to calculating rf_s_ score for individual peptides, rather than producing minigene-specific scores. Where applicable, rf_s_ scores from replicate experiments are calculated as the geometric mean of all measurements. We defined two separate read processing pipelines for the two formats of minigene libraries since WES libraries contain epitopes embedded within an undefined number of possible distinct minigene fragments, WES fragments are generally longer in length than would allow for an accurate paired-end merging step, randomly sheared WES fragments could be cloned into viral vector in one of six possible expression frames (whereas frame is precisely controlled in WPC format), and WPC minigenes are constructed with non-native codon usage.

We have previously described a functional high-throughput screening method to profile cytotoxic T cell (CTL) responses against large libraries of short peptide-coding DNA sequences in a method we term T cell epitope sequencing (Tope-seq) (16). Briefly, this method leverages a granzyme-B (GZMB)-sensitive fluorogenic reporter transgene encoded in target synthetic antigen presenting cells (sAPCs). When mixed with CTLs expressing antigen-responsive TCRs, delivery of GZMB from CTL to target sAPC – a natural response of triggered CTLs – produces a shift in fluorescence properties caused by enzymatic cleavage. The GZMB readout differs from conventional assays of T cell activation-induced markers or target cell lysis as it is a detectable early signal of T cell response spatially located within the target cell that enables precise selection of cells harboring relevant antigens out of bulk populations prior to cell loss due to apoptosis. Hence, it can be applied towards high-throughput functional screening by i) expressing large libraries of DNA-encoded minigenes, delivered as a genome integrated lentiviral vector, into reporter-expressing HLA-matched target sAPC (**Figure 1a**); ii) co-culturing the library of sAPCs with CTL populations of interest; iii) sorting sAPC post co-culture by fluorescence activated cell sorting (FACS) to recover cells containing putatively antigenic minigenes (**Figure 1b**); and iv) characterizing recovered cells by minigene sequencing to reveal peptide-coding sequences responsible for CTL activity (**Figure 1c**). We have validated this approach in the context of randomized minigene libraries screened against primary T cells from peripheral tissue and tumor infiltrates in murine model systems (16). We have also previously implemented the fluorogenic GZMB assay system in human cell lines and demonstrated it in the context of recombinantly expressed TCR and MHC (17).

In this study, our goals were to apply Tope-seq technology to the characterization of TCR autoreactivity. We did this by performing comprehensive functional characterization of a3a [an *ex vivo* affinity-enhanced TCR that previously failed in clinical trials due to cardiotoxicity mediated by unexpected cross-reactivity (18)] and its wild-type thymically-selected counterpart [EB81.103 (19)] against large libraries of potential epitopes representing all possible genome encoded peptides. We show that the therapeutic target epitope, EVDPIGHLY, derived from the MAGEA3 protein was detectable for both TCRs by Tope-seq and the cross-reactive epitope previously determined to be the cause of clinical failure, ESDPIVAQY, derived from the Titin (TTN) protein, was detected in analysis of the affinity-enhanced a3a only. We also report strategies for developing libraries of potential epitopes, contending with background from endogenously expressed proteins, and developing bioinformatic analysis workflows for Tope-seq datasets.

## Results

### Development of minigene libraries spanning self-antigen space

To conduct Tope-seq screening on complete self-antigen minigene libraries, we first needed to develop suitable libraries to express in reporter target cells. In general, we sought to follow the key design principles of balancing the ability to: i) infer the minimal epitopes, derived from full length coding sequences, responsible for T cell reactivity in the HLA allele context under interrogation; ii) manufacture and screen libraries of practical size and complexity; iii) leverage natural cellular machinery to process and present the minimal epitope peptides that would organically be encountered by the TCR under interrogation; iv) avoid biases from coding sequence length, transcript abundance, and protein cellular function in downstream Tope-seq screens.

One approach employed to this end was a whole proteome coding (WPC) minigene synthesis strategy, accomplished by designing *in silico* a set of amino acid sequences representing all possible peptides potentially expressed by the human coding genome. To do this, each protein coding sequence in the human reference proteome was deconstructed *in silico* by extracting tiled amino acid segments and converting these to DNA minigenes by backtranslation. Redundant protein domains observed in protein isoforms or across highly homologous proteins were consolidated prior to tiling to avoid overrepresentation of peptide subsequences in the final fragment library. The resulting set of >5 × 10^5^ minigenes was array synthesized (Twist Biosciences) to yield a single-stranded DNA oligonucleotide pool, which we then cloned into lentiviral transfer plasmid. The prepared WPC library plasmid was characterized by NGS analysis of minigenes amplified directly from starting library plasmid stock, which showed >97% of reads successfully mapped to the designed reference minigenes and >97% of the designed library was represented, indicating a very low incidence of introduced artifacts or sequence dropout (**Supplementary Table 1**).

We then proceeded to use prepared plasmids to generate lentiviral vector and transduce K562 cells, previously engineered to express a HLA-A*01:01/GZMB-FRET reporter gene cassette (referred to as KFRET.A0101 cell line) (17), at a multiplicity of infection (MOI) of 7. We selected a relatively high MOI for library cell line creation because we observed sequence dropout in preliminary library transductions conducted at low MOI (MOI = 0.2) (**Supplementary Table 2**). Hence, we reasoned it would be better to ensure maximal coverage of the designed library by using a high MOI scheme. Although there is risk of passenger minigenes becoming captured in the FRET-shifted minigene population and falsely detected in Tope-seq with a higher MOI library, we anticipated that conducting transduction at a sufficiently large scale to ensure clonal redundancy of each minigene would render it unlikely that any true negative passenger minigenes would repeatedly co-occur with true positive epitopes in the founder clone population. Assessment of WPC minigene libraries post-transduction (**Supplementary Table 3**) indicated each high MOI replicate represented >97% of the designed minigene library.

In parallel to synthetic WPC library development, we undertook an alternative strategy towards the generation of minigene libraries for expression in target reporter cells. We explored primary nucleic acids as source material from which minigene libraries can be built, in this case constructing a comprehensive self-antigen minigene library by using normal human genomic DNA from a mixture of multiple primary healthy donors. The genomic starting material was acoustically sheared and subject to exome capture and adapterization. These steps were performed off-site (Genewiz) using standardized protocols for Illumina library construction and, rather than proceeding with exome sequencing, we interposed minigene vector library cloning and target cell line creation to prepare a Tope-seq-ready exome minigene library. We assessed library diversity by sampling transduced cells and performing NGS analysis of minigene amplicons recovered therein (**Supplementary Table 4**). The results of this analysis indicated that the starting whole exome shotgun (WES) cell library represented >2.5 × 10^7^ unique minigene fragments (or greater than 100X coverage of the human exome). Notably, the diversity of WES format libraries was considerably larger than WPC libraries. This was expected since WES fragments were generated by shearing DNA at random breakpoints and inserted into lentivector cassette in six possible reading frames.

### Comprehensive self-peptide assessment using WPC library

In previous work, we observed FRET-shift signal during co-culture of both EB81.103 and a3a TCR-expressing T cells against KFRET.A0101 cells in the absence of any introduced minigene (17). This suggested the presence of endogenous MAGEA3 protein and potentially other self-antigens in these cells, which we suspected could represent a source of background for library-based screening experiments. Epitopes derived from host cell proteins would render all cells in the target population selectable by FRET-shift FACS, irrespective of the transduced minigenes contained within.

To assess the extent to which this endogenous background would confound the interrogation of exogenous minigene libraries, we performed initial screens of a3a CD8^+^ TCR-T cells with KFRET.A0101 cells containing either WPC or WES minigene library. For each screening experiment, FRET-Shifted and Unshifted target cell fractions were isolated according to the gating strategy illustrated in **Supplementary Figure 1**. Immediately after cell capture, cells recovered from each gate were separately processed for genomic DNA purification. Minigenes integrated in captured genomic DNA samples were recovered by targeted PCR and sequenced. After minigene sequencing, data was processed via Tope-seq analysis pipelines developed specifically for either WPC or WES experiments (**Figure 1c**). Enrichment was measured by computing the relative frequency of each individual library sequence’s normalized count in the Shifted gate sample as a proportion of its total normalized count across both gates (rf_S_). Each rf_S_ score, a sampling proportion of the library population, is normally distributed (**Supplementary Figure 2**). Hence, Z-scores and p-values were produced for each minigene or reference peptide sequence for significance testing. In these initial experiments, we determined positive control epitopes could not be detected from background under these conditions (**Supplementary Figure 3**).

To explore if we could improve detection of test antigens by removing endogenous epitopes derived from host cell proteins in sAPC cell chassis, we used two different strategies. One approach was to use a CRISPR/Cas9 edited version of the KFRET.A0101 cell line, which we had previously developed to incorporate a homozygous deletion of the EVDPIGHLY epitope-coding region within the *MAGEA3* gene (17). We found that this genetic intervention ablated background FRET-shift response in co-culture with EB81.103 TCR-T cells but had no effect on background signal from a3a TCR-T cells (17). We reasoned that this likely was due to the presence of other potential cross-reactive epitopes of a3a endogenously expressed in the K562 cell line. Therefore, as a second approach, we validated in this study an alternative “clean” target cell chassis to use as a host cell line for minigene library screening. We generated sAPC based on 721.221, an MHC-null EBV-transformed B-lymphoblastoid cell line (20), by viral transduction to equip the cell line with HLA-A*01:01 coding sequence and the FRET-reporter transgene (creating the 721FRET.A0101 cell line). Minigene-specific response was distinguishable in initial testing using positive control 721FRET.A0101.MAGEA3^143–202^ cells (**Supplementary Figure 4**), demonstrating that expressed self-epitopes in K562 confounding assessment of the a3a TCR are not also expressed in the 721.221 cell line.

**Figure 2:**
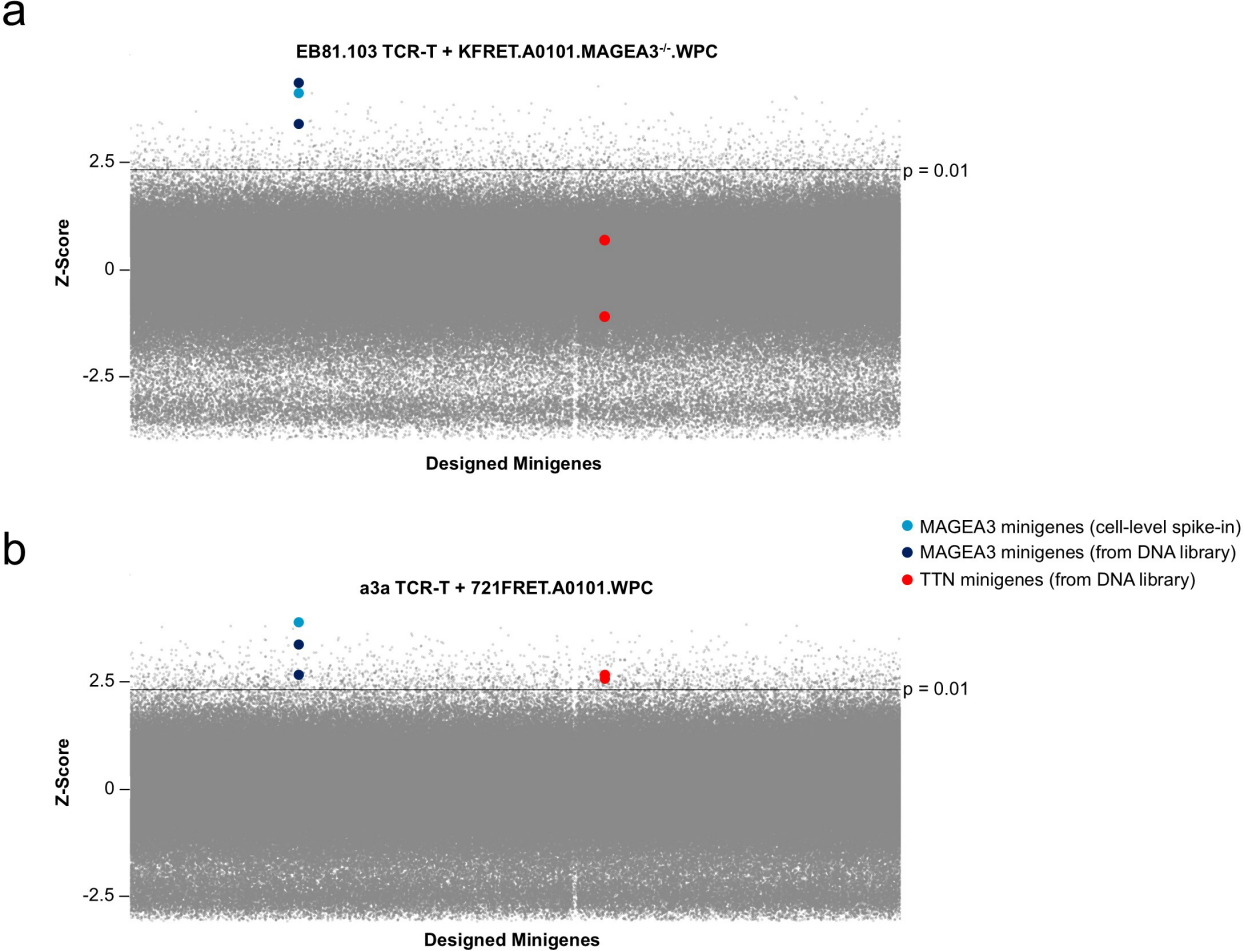
Tope-seq assessment of EB81.103 and a3a TCR against WPC library in clean target chassis. Tope-seq readout from initial test of EB81.103 TCR-T cells co-cultured with CRISPR-edited KFRET.A0101.MAGEA3^-/-^ cells containing WPC library **(a)** or a3a TCR-T cells co-cultured with 721FRET.A0101.MAGEA3^-/-^ cells containing WPC library **(b)**. In both experiments, an effector/target ratio of 1:1 was used to assemble co-cultures. For each screen, >6 × 10^7^ target library cells (spiked with 1.5 × 10^3^ KFRET.A0101.MAGEA3^-/-^.MAGEA3^143–202^ re-introduced minigene cells as an internal control) were incubated with TCR-T cells for either a period of 12 hours (for KFRET cells) or 1 hour (for 721FRET cells) prior to FACS isolation of Shifted and Unshifted cells. Recovered cells were subject to gDNA purification and minigene amplification before Illumina sequencing. Both experiments were conducted in duplicate. The frequency of reads in Shifted relative the normalized read counts in Shifted + Unshifted for each minigene (for panel a) or 9-mer coding genomic locus (for panel b) were computed in each experiment. Geometric mean of minigene scores across both replicates were then calculated and used to calculate Z-scores plotted on y-axes. The control MAGEA3 and TTN epitope-containing minigenes are highlighted according to the figure legend. Significance threshold representing p = 0.01 is indicated on y-axes as a solid line (Z-scores above line are p < 0.01).

We prepared new library cell lines in based on 721FRET.A0101 and KFRET.A0101.MAGEA3*^-/-^* (the native *MAGEA3* gene knockout) reporter lines, focusing on WPC format libraries since they are lower complexity than the WES format libraries, and performed Tope-seq analysis of a3a TCR-T cells and wild-type EB81.103 TCR-T cells, respectively. The results of these Tope-seq screens indicated positive control minigenes could be successfully detected in both experimental conditions. In the EB81.103 TCR screen, both MAGEA3 minigenes containing the EVDPIGHLY epitope were significantly enriched above background (p < 0.001, rank >99.99^th^ percentile) while both TTN minigenes containing the ESDPIVAQY epitope were not significantly enriched (**Figure 2a**). In the a3a TCR screen, the MAGEA3 minigenes were significantly enriched (p < 0.001, >99.8^th^ percentile) while, in this case, both TTN minigenes were also found to be significantly enriched (p < 0.001, rank 99.8^th^ percentile) (**Figure 2b**). These results reflect the findings made in the original clinical case report after severe off-target cross-reactivity was observed *in vivo* and show that comprehensive self-antigen screening of a whole-proteome coding library with Tope-seq would have raised this cross-reactivity as a risk factor during the discovery and development phases of the a3a TCR. This result also confirmed our hypothesis that eliminating response to host cell antigens is sufficient for enabling Tope-seq based assessment of TCRs-of-interest against complex self-antigen libraries.

### Bioinformatic refinement of Tope-seq data by clustering

We applied further bioinformatic analysis to investigate other minigenes enriched above the p < 0.01 threshold in WPC experiments (n = 1,850 in EB81.103 experiment; n = 2,283 in a3a experiment). Our goal was to examine whether putative cross-reactivities arise from common epitope motifs or if multiple epitope clusters emerged for either of the TCRs interrogated, as well as to attempt to estimate signal-to-background in these studies. To do this, we analyzed all 8- to 11-mer peptide sequences embedded in each minigene for both experiments using a clustering approach. As a first step, initial filtering of peptide lists was performed by removing peptides from non-significant (p > 0.01) minigenes. We then determined, for peptides with occurrences in multiple different minigenes, which sequences met the statistical significance threshold more frequently than would be expected due to chance (binomial exact test). After initial filtering, peptide sequences were further filtered by performing peptide-MHC binding prediction using NetMHC 4.0 (21). To avoid introducing undue bias in the analysis, we applied a very low stringency cutoff (rank score < 5). At this stage, for both experiments, 27% of minigenes detected above threshold contained one or more of the peptide subsequences passing filters.

**Figure 3:**
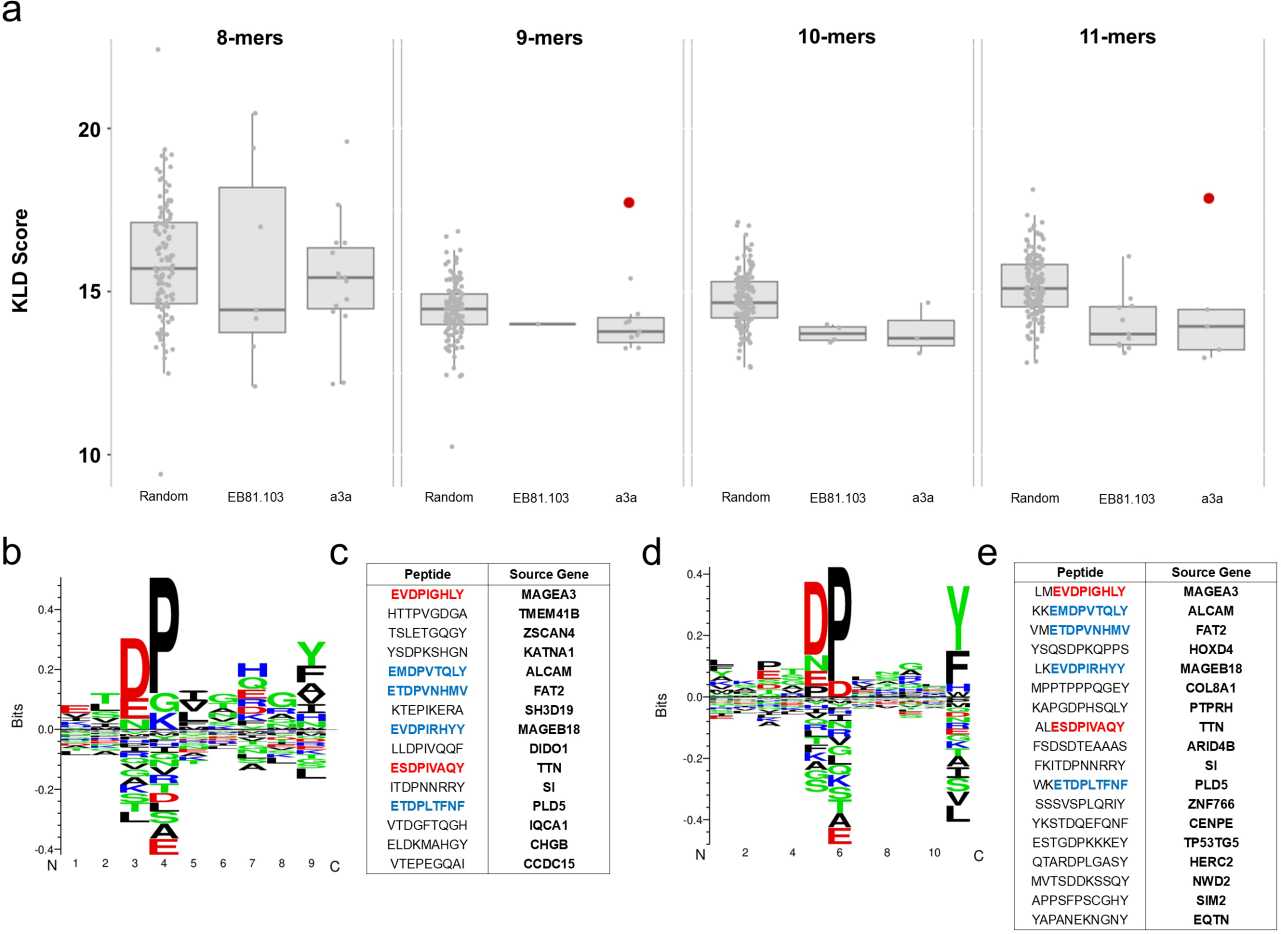
Clusters of similarity emerge among hits from a3a TCR-T WPC screening. **(a)** Minigenes in EB81.103 TCR-T + KFRET.A0101.MAGEA3^-/-^ and a3a TCR-T + 721FRET.A0101 WPC library experiments were broken into k-mers of sizes ranging from 8–11 amino acids and filtered on Z-score threshold and NetMHC 4.0 binding (using a relaxed threshold of rank < 5). Filtered peptide lists were used as input sequences for GibbsCluster 2.0, testing at various K-means clustering ranging from 1–15 clusters. For each experiment of each peptide size, the K-means regime producing the highest average Kullback-Leibler Divergence (KLD) score was carried forward for further analysis (individual clusters plotted here with interquartile boxplot overlay). For significance testing, clusters from each solution were compared to a baseline distribution generated from clustering randomized peptides (all filtered to meet rank score < 5 in NetMHC 4.0). The random baseline represents cluster scores from the top performing solution in ten iterations of random peptide clustering and was found to be approximately normally distributed by Kolmogorov-Smirnov test. Only two clusters (highlighted in red) were found to be statistically significant compared random peptide background (p < 0.01 by one-sample Z-test). These clusters were found in the 9-mer and 11-mer analysis of the a3a TCR-T experiment. Motifs of the significant clusters are shown in **(b, d)** and the core alignment peptides for each cluster are shown in **(c, e)**, respectively. Amino acids highlighted in red represent known test peptides from MAGEA3 and TTN. Amino acids highlighted in blue represent peptides previously observed in other analyses of the a3a TCR (23). Non-highlighted represent peptide hits not previously observed in orthogonal studies. Peptides, ranked by rf_s_ score in Tope-seq experiments, are displayed with source human genes.

Peptides were then grouped using GibbsCluster 2.0 (22), an algorithm for simultaneous alignment and clustering based on a Gibbs sampling approach for deconvoluting motifs in complex sets of sequences (processed sequence lists from each step can be found in **Supplementary Data**). Motif detection was performed by producing a baseline distribution of motifs from randomly generated peptides and conducting significance testing of GibbsCluster scores from each experiment-derived motif against the distribution of scores from baseline random peptide motifs (**Figure 3a**). Overall, only two motifs emerged with statistically significant GibbsCluster scores, both arising from the a3a TCR dataset (**Figures 3b, d**). The two emergent motifs were derived from different size peptide analyses but contained notable overlap in the core peptides used to generate them. Both motifs contained the known positive epitopes from MAGEA3 and TTN, as well as additional reactivities that had been previously reported in prior studies (23). In the EB81.103 TCR dataset, in contrast to a3a, the positive MAGEA3 epitope was not found to cluster with any other putative peptide hits, implying that cluster membership is not a strict prerequisite of immunogenicity by this analysis. This result is consistent with the expectation that wild-type thymically selected TCR would have naturally have higher precision than affinity engineered variants. This also suggests the affinity maturation undertaken to convert the EB81.103 TCR to a3a had the effect of increasing plasticity of TCR response from one precise epitope to a larger grouping of highly similar peptides, as opposed to creating multiple distinct outgroups of reactivity.

The statistically significant clusters detected in our a3a dataset contained additional reactivities that were not previously reported and may represent newly discovered self-epitopes of the a3a TCR (**Figures 3c, e**), some of which have high tissue-specific expression in Human Protein Atlas (24) reference data (**Table 1**) and may also potentially be clinically relevant. Some putative WPC hits from a3a screening were also shown to be expressed in Human Protein Atlas data for the K562 cell line. These potential K562-specific antigens are expressed at relatively lower levels in 721.221 cells (25), providing evidence supporting the hypothesis that some of the detected putative epitopes from this experiment were contributing to background signal in K562 chassis-based library screens.

**Table 1:** Tissue- and K562 cell line-specific expression of putative self-antigen hits from a3a TCR-T WPC screen.

Peptide	Source gene	Minigene enrichment (Z-score)	Human protein atlas tissues (Consensus nTPM)	Human protein atlas – K562
EVDPIGHLY LM**EVDPIGHLY**	*MAGEA3*	3.90 3.39	Testis (7.8)	nTPM = 134.3; not detected by mass spectrometry
HTTPVGDGA	*TMEM41B*	3.34	Liver (18.2)	**nTPM = 47.7; detected by mass spectrometry**
TSLETGQGY	*ZSCAN4*	3.19	Stomach (2.4)	Not detected
YSDPKSHGN	*KATNA1*	3.14	Testis (43.8)	**nTPM = 41.8; detected by mass spectrometry**
EMDPVTQLY KK**EMDPVTQLY**	*ALCAM*	3.12 2.70	**Parathyroid Gland (424.3)**	Not detected
KTEPIKERA	*SH3D19*	3.07	Adipose (68.3)	Not detected
ETDPVNHMV VM**ETDPVNHMV**	*FAT2*	2.94	**Cerebellum (226.4)**	Not detected
YSQSDPKQPPS	*HOXD4*	2.82	Epididymus (27.3)	Not detected
EVDPIRHYY LK**EVDPIRHYY**	*MAGEB18*	2.80	Testis (0.7)	Not detected
MPPTPPPQGEY	*COL8A1*	2.79	Heart Muscle (37.8)	Not detected
LLDPIVQQF	*DIDO1*	2.76	Skin (38.3)	**nTPM = 36.5; detected by mass spectrometry**
KAPGDPHSQLY	*PTPRH*	2.74	Small Intestine (66.7)	nTPM = 22.5; not detected by mass spectrometry
ESDPIVAQY AL**ESDPIVAQY**	*TTN*	2.67 2.65	**Skeletal Muscle (887.9)** **Heart Muscle (161.7)**	nTPM = 1.2; detected by mass spectrometry
FSDSDTEAAAS	*ARID4B*	2.56	Bone Marrow (32.7)	nTPM = 15.8; not detected by mass spectrometry
ITDPNNRRY FK**ITDPNNRRY**	*SI*	2.49	**Duodenum (530.0)** **Small Intestine (435.9)**	Not detected
ETDPLTFNF WK**ETDPLTFNF**	*PLD5*	2.48	Choroid Plexus (26.8)	Not detected
YKSTDQEFQNF	*CENPE*	2.47	Bone Marrow (9.9)	nTPM = 27.8; not detected by mass spectrometry
SSSVSPLQRIY	*ZNF766*	2.47	Testis (23.0)	nTPM = 12.6; not detected by mass spectrometry
ESTGDPKKKEY	*TP53TG5*	2.46	Testis (17.3)	Not detected
MVTSDDKSSQY	*NWD2*	2.45	Cerebral cortex (3.9)	Not detected
QTARDPLGASY	*HERC2*	2.45	Skeletal Muscle (37.8)	**nTPM = 16.8; detected by mass spectrometry**
APPSFPSCGHY	*SIM2*	2.43	Kidney (59.6)	Not detected
YAPANEKNGNY	*EQTN*	2.41	Testis (36.7)	Not detected
VTDGFTQGH	*IQCA1*	2.39	Choroid Plexus (48.8)	Not detected
ELDKMAHGY	*CHGB*	2.37	**Adrenal Gland (3361.4)** **Pituitary Gland (1387.3)** **Cerebellum (677.1)**	nTPM = 0.4; detected by mass spectrometry
VTEPEGQAI	*CCDC15*	2.34	Testis (10.2)	nTPM = 5.0; not detected by mass spectrometry

9-mer and 11-mer peptides, comprising the core alignments of detected epitope motifs derived from Tope-seq analysis of a3a TCR-T, were cross-referenced with respect to their source antigenic protein to the Human Protein Atlas dataset. Peptides with complete overlap were grouped together for analysis. The source genes of each putatively immunogenic epitope and Z-scores from library minigenes containing epitopes are shown. For each, the consensus normalized transcript per million (nTPM) values for the tissue type with the highest reported transcript level were retrieved from the Human Protein Atlas and shown here. Multiple tissue types were reported (in bold) for cases where transcript level was higher than expression level of *TTN* in heart muscle, which was determined to be clinically relevant in the original case report. Expression level of motif core alignment peptides was also investigated for K562 data included in the Human Protein Atlas cell line repository. The nTPM values, where applicable, are shown below as well as presence or absence scoring at the protein level as determined by mass spectrometry.

Bold emphasis denotes common subsequences.

### Biopanning as a strategy for overcoming endogenous reactivities

Our strategies for deleting endogenous epitopes by genome editing or developing alternative clean target cell chassis to prepare a suitable library host for Tope-seq based self-antigen assessment were effective in this study. However, we considered that these options may not always be available, particularly in cases where putative self cross-reactivities are unknown or are in essential genes that cannot be knocked out or otherwise avoided. Therefore, in addition to exploring engineered or alternative target cell lines for avoiding background from endogenous antigens, we sought to investigate whether this background could be overcome by iteratively re-screening selected minigenes to enrich epitopes progressively, analogous to biopanning techniques used in phage-display technologies (26). Because of the large diversity of unique minigenes in WES libraries, as compared to WPC, we performed testing of biopanning in these libraries anticipating that significant bottlenecking of the library diversity between first and second round experiments would enable us to see enrichment effects clearly. Our hypotheses were twofold. We first expected that re-screening leftover Shifted gate amplicon produced during NGS library preparation from a 1^st^ round experiment would result in increasing enrichment scores of known positive epitopes. We also expected that most peptides with significant enrichment scores from the first round would be undetected or non-significant in the second round.

**Figure 4:**
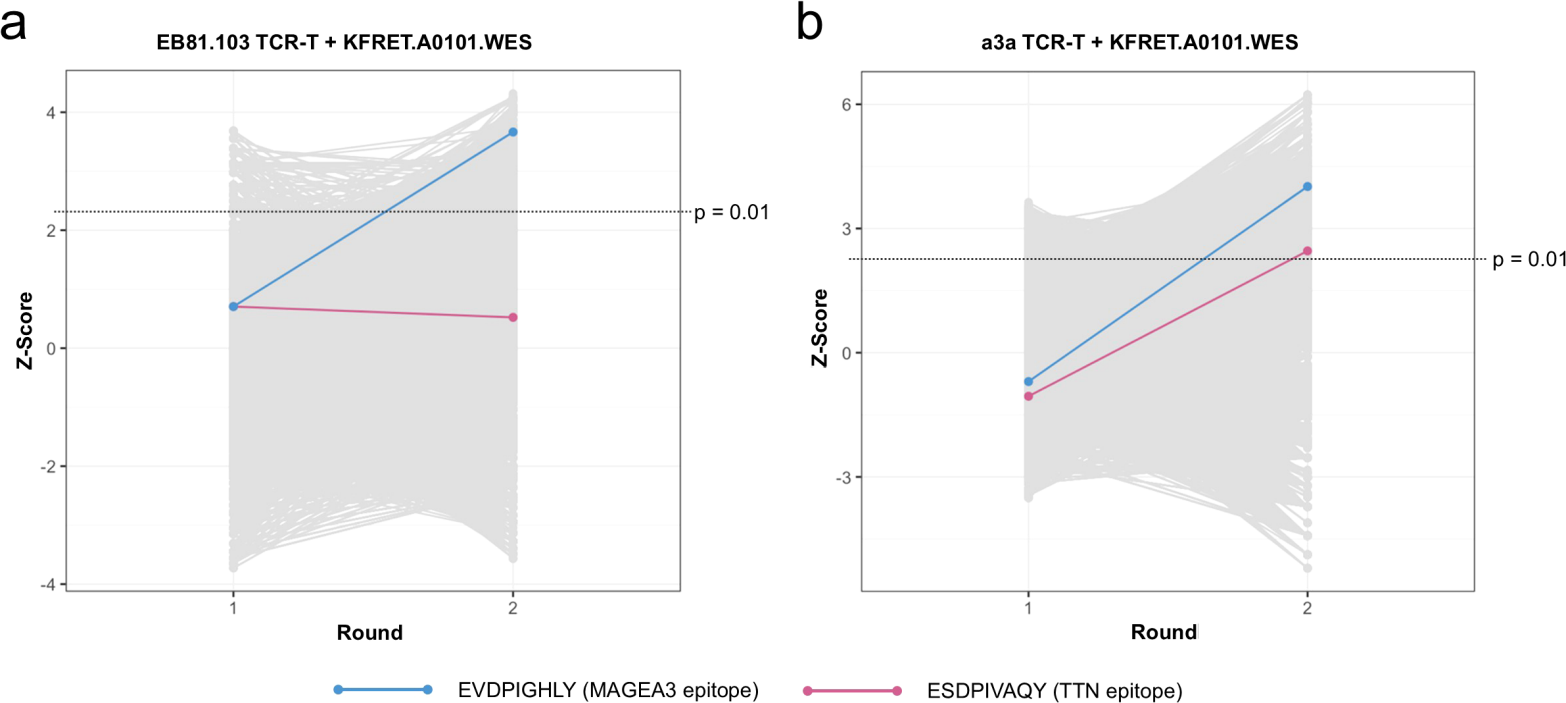
Enrichment observed in 2^nd^ round panning screen of WES library. Tope-seq readouts from 1^st^ and 2^nd^ round screens of **(a)** EB81.103 TCR-T cells and **(b)** a3a TCR-T cells against non-CRISPR edited KFRET.A0101 cells containing WES library (for 1^st^ round experiments) or respective re-cloned panning library (for 2^nd^ round experiments). In both experiments, an effector/target ratio of 1:1 was used to assemble co-cultures. For each screen, >6 × 10^7^ target library cells were incubated with TCR-T cells for a period of 12 hours prior to FACS isolation of Shifted and Unshifted cells. Recovered cells were subject to gDNA purification and minigene amplification before Illumina sequencing. The frequency of in-frame reads spanning each 9-mer coding genomic locus in Shifted relative the normalized read counts in Shifted + Unshifted were computed and used to calculate Z-scores plotted on y-axes. Each peptide scored in the 1^st^ and 2^nd^ rounds are plotted as a grey point in each group and connected between rounds by a line to indicate increase or decrease after panning. The control minimal epitopes from MAGEA3 and TTN are highlighted according to the figure legend. Significance threshold representing p = 0.01 is indicated on y-axes as a solid line (Z-scores above line are p < 0.01).

We constructed 2^nd^ round WES libraries by re-adapterizing Shifted gate minigenes from 1^st^ round screens of both TCRs for cloning by PCR and inserted them into library expression lentiviral transfer plasmid. Cloned 2^nd^ round libraries were used to generate lentiviral vector and create library cell lines in non-CRISPR edited KFRET.A0101 cell chassis. Sampling the prepared 2^nd^ round libraries for NGS QC revealed that both libraries contained 1-2 × 10^6^ unique minigenes (representing 4-8% of the minigenes contained in the original library) (**Supplementary Table 5**). Tope-seq experiments were conducted for TCR-T cell preparations containing either EB81.103 or the a3a TCR against their respective 2^nd^ round library. Our results indicated that, in the EB81.103 TCR experiment, the EVDPIGHLY epitope from MAGEA3 was significantly enriched above background (p < 0.001, rank >99.99^th^ percentile) after 2 rounds while the ESDPIVAQY epitope from TTN was not significantly enriched (**Figure 4a**). In the a3a TCR screen, the MAGEA3 epitope was significantly enriched (p < 0.001, rank >99.9^th^ percentile) while the TTN epitope was also found to be significantly enriched (p < 0.01, ranked >95^th^ percentile) after 2 rounds (**Figure 4b**). These results were similar to what we observed in the analysis of WPC library in clean background cells, indicating that iterative re-screening, or biopanning, can also be deployed as a tool, either independently or potentially in conjunction with chassis editing strategies, for contending with background arising from naturally occurring antigens in live host sAPC.

We next sought to investigate broad patterns among other enriched peptides in the dataset. To this end, we scored peptides from 8-mer, 10-mer and 11-mer reference window settings. Strikingly, we noted that for all analyses, there were >10-fold more peptides meeting significance threshold after both rounds in the a3a experiment than in EB81.103 (**Table 2**), perhaps reflecting the highly promiscuous nature of the a3a TCR relative to its wild-type, thymically-selected counterpart. Repeating the filtering and clustering analysis described above for the WPC experiments, we were unable to derive new statistically significant peptide motifs from the WES library format. Since the 2^nd^ round WES panning libraries still represented a substantially larger number of unique minigenes than are present in the WPC library, we considered that additional panning would be necessary to detect clusters of reactivity by this method. We also observed that most peptides (>93% for all analyses) meeting the p-value cutoff in the 1^st^ round were not re-discovered in the 2^nd^ round, supporting our hypothesis that the panning approach is useful for removing background. However, a larger proportion of 1^st^ round hits were re-discoverable in the 2^nd^ round than would be expected due to chance (1.4% and 6.3% for EB81.103 and a3a 9-mer analyses, respectively; p < 0.001 by one-tailed exact binomial test). Additionally, although the dataset was approximately evenly split between peptides increasing in score (incliners) and those decreasing between rounds (decliners), a significant proportion of the incliner population was found to meet the cutoff in 2^nd^ round screening for both experiments (1.6% and 13.3% for EB81.103 and a3a 9-mer analyses, respectively; p < 0.001 by one-tailed exact binomial test), which provided evidence that positive enrichment of immunogenic epitopes was occurring.

**Table 2:** Summary of putative enrichment dynamics in two rounds of Tope-seq biopanning of WES self-antigen library.

Summary Metric	EB81.103				a3a			
	8mers	9mers	10mers	11mers	8mers	9mers	10mers	11mers
# Peptides 1^st^ rd.	5,299,213	5,161,203	5,023,895	4,887,440	5,140,672	5,005,841	4,871,757	4,738,480
# Peptides 2^nd^ rd.	1,673,140	1,620,446	1,568,225	1,516,499	1,739,540	1,682,888	1,626,736	1,571,267
% Peptides with 1^st^ rd. score < 2^nd^ rd. score (incliners)	41.9	42.0	42.1	42.1	50.6	50.8	50.9	51.1
% Peptides with 1^st^ rd. score > 2^nd^ rd. score (decliners)	58.1	58.0	57.9	57.9	49.4	49.2	49.1	49.9
% Sig. peptides (p < 0.01) from 1^st^ round re-discovered in 2^nd^ rd	1.8	1.4	1.3	1.2	7.3	6.3	5.5	5.0
# Sig. peptides (p < 0.01) 2^nd^ rd.	10,823	10,635	10,437	10,283	124,458	120,367	116,284	112,250
Incliners as % of 2^nd^ rd. sig. peptides	99.4	99.3	99.3	99.2	94.5	94.5	94.4	94.4
% Incliners n.s. in 1^st^ rd. & sig. in 2^nd^ rd.	1.53	1.55	1.57	1.60	13.3	13.3	13.3	13.2

Experiment results from both the EB81.103 (wild-type) and a3a (engineered) TCR variants were processed for all peptide sizes ranging from 8–11 amino acids in length. Shown, for each experiment and size analysis, are the number of peptides detected in each round (as well as the number of peptides meeting significance threshold after two rounds), the percentages of peptides that increased or decreased in rf_S_ score between rounds, the percentages of significant peptides from the 1^st^ round also found to be significant in the 2^nd^ round, the percentage of significant peptides from round 2 that increased in rf_S_ score between rounds, and the percentage of non-significant peptides from the 1^st^ round that became significant after the 2^nd^ round.

## Discussion

A key lesson taken from the original case report of the catastrophic failure of the a3a TCR (27) is the urgent need for functional assessment of engineered therapeutic TCR against the widest panel of possible self-antigens to rule out dangerous off-target cross-reactivities early in therapeutic development. In this study, we used the a3a TCR as a test case to evaluate the utility of Tope-seq in directly screening all peptides encoded in the genome for their potential to elicit autoreactivity.

Currently. the most common *in vitro* functional assay for probing TCR cross-reactivity is based on peptide-pulsing target cells with pools of partially randomized peptide to deduce putatively important residues necessary for TCR triggering (23, 28–30). The main drawbacks of this technique are that it i) does not account for the inherent biases present in the antigen processing and presentation machinery used to generate natural pMHC ligands in cells; ii) interrogates TCR in the context of supraphysiological abundance of pMHC ligand; iii) is indirect and may not be able to identify cross-reactivities which do not have significant sequence similarity to the index peptide or represent distinct epitope clusters (31); and iv) requires a different custom set of peptide libraries to be generated for the study of each new index peptide. In contrast, we present Tope-seq as a complementary approach for functional cross-reactivity assessment that preserves natural expression, processing, and presentation of library peptides. Genetically encoded cell libraries containing a defined panel of potential epitopes, such as all possible self-antigens, also provide an advantage as they can be directly searched without the need to infer cross-reactivity based on results from degenerate peptide. Additionally, the minigene library system used in conjunction with Tope-seq assay readouts can be flexibly re-used in studies of different TCR, HLA, or target peptide contexts without re-synthesis.

To validate the Tope-seq approach to the assessment of comprehensive self-antigen libraries, it was essential in this study, as in any functional live cell assay, to consider non-specific background from endogenous peptides in the target cell chassis. We found that epitopes of the test TCR derived from naturally expressed host cell proteins were indeed a confounding factor in analyzing minigene library-derived epitopes and we deployed solutions such as CRISPR/Cas9 removal of known expressed epitopes and adaptation of alternative cell chassis lines to address this issue. Though successful in the present study, these solutions are not universal since endogenous background epitopes may not be known or may exist in an indispensable gene. We, therefore, also sought to overcome endogenous peptide background by performing iterative biopanning through minigene libraries, progressively enriching hits on each round. We did this by directly re-cloning and re-expressing primary DNA from captured minigenes for second-round Tope-seq analysis, which was an effective approach for increasing enrichment in known epitopes even in target cell chassis where endogenous epitopes were present.

We also noted several other putatively enriched sequences in our WPC and WES self-antigen Tope-seq experiments. Using bioinformatic sequence clustering-based methods, we inferred some of these may likely be additional epitopes of the a3a TCR on the basis of sequence similarity with known epitopes. However, since it is possible for cross-reactive epitopes of the same TCR to differ substantially in sequence identity (6), it was still unclear what proportion of the threshold minigene hits contained *bona fide* TCR epitopes and what proportion were falsely identified. In the biopanning workflow tested using WES format libraries, we observed increase in signal from the known test epitopes as well as removal of many first round hits not detected in second round screening. Hence, we propose that our new biopanning workflows for improving signal-to-noise will provide a feasible route toward unambiguously selecting immunogenic epitopes from future large library screens using relatively few replicate experiments.

The selection of method for minigene library production is another important consideration in the development of Tope-seq screening campaigns. The flexible and modular nature of the Tope-seq system enables a variety of DNA sources to be accessed as a source of minigene library. In our work, we describe two alternative pathways for producing panels of peptide-coding sequences to be interrogated by TCRs-of-interest. In one iteration, we used a defined, computationally designed, array-synthesized version of a self-antigen library while in the other, genomic DNA from primary samples was sheared and exome-captured. We envision that a variety of minigene library types could be developed and deployed in Tope-seq screening campaigns depending on specific experimental considerations. In **Table 3**, we contemplate the advantages and disadvantages across the spectrum of minigene library formats.

**Table 3:** Multiple routes are available for constructing genetically encoded minigene libraries to screen by Tope-seq.

	Synthesized DNA from reference proteome	Exome-captured genomic DNA fragments	cDNA fragments	Whole-genome DNA fragments	Randomly generated oligos
**Complexity**	Low/Medium (costly to scale-up).	Medium	High	Very high	Extremely high (very difficult to comprehensively search)
**Gaps**	Polymorphism/strain variation. Non-coding regions.	Polymorphism/strain variation. Exon-exon junctions. Non-coding regions.	Polymorphism/strain variation. Rare transcripts. Tissue-specific transcripts.	Polymorphism/strain variation. Exon-exon junctions.	Theoretically minimal.
**Background**	Artifacts from synthesis errors.	Flanking intronic sequence. Out-of-frame peptides.	5’-/3’-UTRs. Out-of-frame peptides. Highly abundant transcripts.	Non-coding regions. Out-of-frame peptides.	Theoretically maximal.
**Fidelity**	Limited by DNA synthesis coupling efficiency (~1 error per 3 × 10^3^ bp).	High, only limited by PCR error rate.	Limited by reverse transcriptase error rate (~1 error per 2 × 10^3^ bp).	High, only limited by PCR error rate.	Very high, only limited by biases in coupling efficiency.
**Advantages**	Expression frame can be strictly controlled. No primary tissue required.	Cost-efficient. No up-front sequencing data required.	Cost-efficient No up-front sequencing data required. More coverage than exome-only library.	Cost-efficient No up-front sequencing data required. More coverage than transcriptome-only library.	Cost-efficient (pooled synthesis) No up-front sequencing data required. No primary tissue required. Most unbiased format.
**Use cases**	Neo-antigen panels. Microbial ORF libraries. Fine epitope mapping of individual proteins. Self-antigen libraries.	Unbiased primary tumor isolate libraries. Minor histocompatibility antigen discovery. Cryptic epitopes. (32)	Unbiased primary tumor isolate libraries. Minor histocompatibility antigen discovery Cryptic epitopes (32)	Unbiased primary viral/microbial isolate libraries. Cryptic epitopes (32)	Training data for *in silico* models.

Two major methods for deriving minigene libraries for Tope-seq analysis were described in this work: synthesized DNA from reference proteome and exome-captured genomic DNA fragments. However, applying the same principles of library construction, screening, and data analysis demonstrated here, other library formats could be contemplated, including cDNA fragment libraries, whole-genome fragment libraries, or synthesized DNA from degenerate, randomized/semi-randomized sequence design. Each of these formats carry specific advantages and disadvantages with respect to the search space afforded, potential gaps or missing sequences inherent, sources of possible background, and issues with DNA sequence fidelity. As a result, we propose a continuum of use cases in which different library construction methods may be optimal for distinct settings in which investigators would seek to test TCR clones or populations against large sets of possible epitopes.

In conclusion, we have described the latest advances in our Tope-seq methodology, including the development of comprehensive synthesized and captured DNA-coded self-antigen minigene libraries, sAPC chassis cell line engineering, and techniques for iterative functional minigene re-screening. We show that this technology has significant potential utility in assessing functional off-target and dangerous cross-reactivity against healthy tissues in therapeutic TCR lead candidates, though additional validation is needed. We expect the application of function-based high-throughput screening to early discovery in therapeutic TCRs will enable effective de-risking, triage, and re-engineering of lead candidate molecules prior to resource intensive pre-clinical analysis.

## Methods

### Plasmid DNA propagation and isolation

NEB Stable Competent *E. coli* (NEB) was used for the propagation of DNA unless otherwise specified. Bacterial transformations were performed according to manufacturer protocol. *E. coli* was grown in LB broth at 30°C, shaking at 250 rpm. For solid medium, LB broth was supplemented with Bacto agar (1.5% [w/v]; Difco). Media were further supplemented with 100 μg/mL carbenicillin or 50 μg/mL kanamycin as appropriate. Plasmid DNA was isolated using Invitrogen PureLink HiPure Plasmid Filter Maxiprep Kit. All synthesized DNA was produced by Integrated DNA Technologies unless otherwise specified. All cloning enzymes were sourced from New England Biolabs unless otherwise specified. Sanger sequencing verification was performed at all intermediate steps by Genewiz.

### DNA fragment library preparation

WPC design was performed using a custom Python pipeline to produce sets of overlapping non-redundant 60 amino acid fragments (with a minimum 30 amino acid overlap) generated from individual protein sequences from UniProt proteome accession number UP000005640. Extracted amino acid sequences were back-translated using randomized codon usage and designed with Illumina adapter sequences flanking proteome-coding minigenes to facilitate cloning and downstream sequencing (library design.fasta file available at https://github.com/DrGovindaSharma). We chose to use non-native codons for producing minigene sequences to mitigate concatemerization of overlapping single-stranded DNA in downstream PCR-based library cloning in plasmid vector. The designed set of DNA sequences (216bp × 535,815 sequences) was synthesized on array-based platforms by Twist Biosciences and delivered as a pool of single-stranded DNA. Library was rendered double stranded and amplified by PCR with Phusion polymerase (1 ng, 30 cycles) prior to being used as input for library cloning procedures. For WES library construction, 1 µg of human genomic DNA (Promega cat. #G3041, lot #0000387521) was shipped to Genewiz for Illumina whole exome-sequencing library preparation only. Prepared exome library was shipped back from Genewiz and PCR-tailed (15 ng, 25 cycles) to amplify library fragments and prepare for cloning into lentiviral transfer plasmid. For WES second-round libraries, 100 ng of genomic DNA sampled from Shifted gate cells captured in first round experiments was subject to PCR amplification and re-cloning. The PCR primers used for all library constructions described here were composed of universal Illumina adapter binding regions tailed with I-SceI/PI-SceI endonuclease sites (F: 5’-TAGGCAAATCTAGGGATAACAGGGTAATTACGACGCTCTTCCGATCT-3’ and R: 5’-ATCGATACATCCATTTCATTACCTCTTTCTCCGCACCCGACATAGATCGTGTGCTCTTCCGATCT-3’) and were HPLC- and PAGE-purified (IDT).

### Construction of HLA plasmids

Lentiviral transfer plasmids were derived from pCCL-c-MNDU3-X backbone (Addgene #81071). To produce an acceptor cassette for HLA allele sequences, a custom synthesized cassette (configured as HLA receiver stuffer-P2A-ECFP-GZMB cleavage substrate-EYFP) was inserted by endonuclease cloning between the BamHI/XhoI restriction sites in the original plasmid (BamHI site ablated during this step) to yield the pHLAI-FRET backbone. Coding sequence for the HLA-A*01:01 allele-of-interest was custom synthesized and cloned into designated HLAI positions via NheI/MluI restriction cloning (restriction sites introduced in acceptor plasmid construction).

### Construction of minigene plasmids

Lentiviral transfer plasmids were derived from pCCL-c-MNDU3-X backbone (Addgene #81071). To produce an acceptor cassette for individual minigenes or minigene libraries, a custom synthesized cassette (configured as Minigene receiver stuffer-IRES-mStrawberry) was inserted by endonuclease cloning between the BamHI/XhoI restriction sites in the original plasmid (BamHI site ablated during this step) to yield the pMGI backbone. Synthesized minigenes and captured DNA fragment minigenes were PCR amplified with primers annealing to proximal adapter sequence flanking minigene fragments and tailed with I-SceI/PI-SceI meganuclease recognition sites. Individual minigenes were cloned into pMGI by standard I-SceI/PI-SceI restriction cloning. Minigene libraries were cloned into pMGI using a specialized multistep digestion/ligation procedure to increase library cloning efficiency and fidelity. Briefly, this procedure consists of i) PI-SceI/PmeI-linearized pMGI ligated under high DNA concentration T4 ligation conditions with PI-SceI-treated library fragments, ii) secondary digestion of linear pMGI/library intermediate with I-SceI, and iii) second low-concentration ligation of I-SceI treated linear pMGI-library plasmid to re-circularize final library vector. Prepared pMGI-library circular plasmids were bacterially expanded by transformation of MegaX DH10B T1^R^*E. coli* (ThermoFisher) by electroporation (Bio-rad GenePulser II) according to manufacturer protocol and 18-hour, 37°C outgrowth on 576 cm^2^ solid agar surface prior to colony scraping and plasmid isolation. Further detail on high-throughput cloning protocol can be found in Appendix A of ref (33).

### Construction of TCR plasmids

Lentiviral transfer plasmid was derived from the pCCL-c-MNDU3-X backbone (Addgene #81071). To produce an acceptor cassette for TCR, a custom synthesized cassette (configured as a TCR receiver stuffer-P2A-mStrawberry) was inserted by endonuclease cloning between the BamHI/XhoI restriction sites in the original plasmid (BamHI site ablated during this step) to yield the pTCR backbone. EB81.103 and a3a TCR cassettes were synthesized as TCRα-T2A-TCRβ fragments and were cloned into pTCR via BamHI/EcoRI restriction cloning (restriction sites introduced in acceptor plasmid).

### Mammalian cell culture

All cell cultures were maintained in RPMI-1640 supplemented with 2 mM GlutaMAX, 1 mM sodium pyruvate, 50 μM β-mercaptoethanol, 10 mM HEPES, 100 U/mL penicillin, 100 U/mL streptomycin, 1X MycoZap™ Prophylactic (Lonza) and 10% heat-inactivated fetal bovine serum. Culture media and supplements were all sourced from Gibco unless otherwise indicated. K562 and HEK-293T were sourced from ATCC; 721.221 cells were a kind gift from the Judy Lieberman lab (Boston Children’s Hospital). All cell cultures were maintained at 37°C and 5% CO_2_ atmosphere. K562 based cell lines were started at 2 × 10^5^ cells/mL and subcultured every 4 days by diluting cells 1/10. HEK-293T were passaged every 4 days by trypsinizing, washing 1X with PBS, and re-seeding cells at 4 × 10^6^ cells/flask in T-75 format. 721.221 cell lines were started at 1 × 10^5^ cells/mL and subcultured every 3 days by diluting cells 1/10. Cell counts were performed using an EVE automated cell counter. All cell lines were certified to be free of mycoplasma contamination using the Venor GeM Mycoplasma Detection Kit (Sigma) prior to experimental data collection. All mammalian cell cultures were incubated at 37°C and 5% CO_2_.

### Virus production

Lentiviral vectors were produced by combining 180 µg of each transfer plasmid with 162 µg of pCMV-ΔR8.91 and 18 µg of pCMV-VSV-G plasmids. These DNA mixes were incubated with 18 mL OptiMEM and 1 mL of TransIT-LT1 reagent (Mirus) for 30 minutes at room temperature. To 12 × T-75 culture flasks containing between 16–24 million HEK-293T cells, old media was removed and replaced with 10 mL fresh, pre-warmed media and 1.5 mL of transfection mix per plate. Media was again replaced 18 hours post-transfection with 10 mL of pre-warmed fresh media. Viral supernatant was then collected at 48 hours post transfection (replacing with 10 mL pre-warmed fresh media) and 72 hours post-transfection. To concentrate virus, pooled supernatants were ultracentrifuged (100,000 RCF, 90 minutes, 4°C). Viral pellets were resuspended at 4°C overnight in 1 mL OptiMEM. Titers of viruses were determined by testing 2, 4, 8, 16, 32 or 64 μL of 10X diluted virus on 1 × 10^5^ K562 cell/well in a final volume of 500 μL of media in 24-well format. Transduction efficiency was determined by measuring the % of fluorescent cells detected in flow cytometry. Values were used to determine K562-infecting units (KIU) per µL of undiluted virus concentrate.

### TCR-T cell production

Peripheral blood leukocytes used as a source of primary T cells were obtained from Stemcell Technologies. We report, in line with BRISQ Tier 1 recommendations that human biospecimens used in this study were leukapheresis products isolated from live normal human donors. Volunteer donors were screened for complete blood count, serum proteins, hematocrit, and confirmed negative for HIV-1, HIV-2, hepatitis B and C prior to procedure. Blood was drawn directly from arm vein into leukapheresis machine, and the isolated leukocyte fraction was collected directly into sample bag containing acid citrate dextrose solution An anticoagulant and stored/shipped at 0-4°C. On receipt, leukopaks were processed using a Ficoll-Hypaque density gradient centrifugation and ACK buffer red-blood cell lysis procedure, both according to manufacturer provided protocols. The resulting isolated PBMCs were aliquoted into individual vials of 1 × 10^7^ cells and cryopreserved in liquid nitrogen vapor phase in heat-inactivated fetal bovine serum + 10% DMSO. Prior to each Tope-seq experiment, ten vials of PBMC (~10^8^ cells total) were thawed and FACS-sorted to isolate CD8^+^ CD4^-^ CD56^-^ cells (using BV785-conjugated anti-CD8 antibody, clone SK1 (Biolegend); PerCP-Cy5.5-conjugated anti-CD4 antibody, clone RPA-T4 (Biolegend); and FITC-conjugated anti-CD56 antibody, clone HCD56 (Stemcell)) and rested for 48 hours in complete RPMI + 300 IU/mL human IL-2 (Stemcell) at a cell density of 10 × 10^6^ cells/mL. Simultaneous T-cell transduction and activation was performed by counting CD8^+^ T cells after the rest period, combining cells with 30 KIU/live cell of TCR-encoding lentiviral vector, and transferring the cell/lentivirus mixture to wells of a non-TC treated, flat-bottom 96-well plate pre-coated with LEAF-purified anti-CD3 antibody, clone OKT3, and anti-CD28 antibody, clone 28.2 (eBioscience). Cells were stimulated for 20–24 hours on coated wells at 37°C and 5% CO2 atmosphere. After the transduction/stimulation period, cells were removed from coated wells, diluted to 1 × 10^3^ cells/mL in complete RPMI + 300 IU/mL hIL-2, and plated in U-bottom 96-well plates. Media was 50% changed every 4 days and used in co-culture experiments 11–14 days after stimulation.

### Creation of HLA-FRET sAPC lines

K562 cell lines were transduced with viral vector produced using pHLAI-A0101-P2A-FRET plasmid at an MOI of 1 KIU/cell. 721.221 cell lines were transduced with viral vector produced using pHLAI-A0101-P2A-FRET plasmid at an MOI of 3 KIU/cell in serum-free OptiMEM for 24 hours prior to returning cells to complete media. Transductions were performed by placing 1 × 10^5^ cells/well in a final volume of 500 μL of media in a single well of 24-well format for 48 hours before resuming normal culture technique to expand. Purified HLA-expressing K562 and 721.221 (KFRET.A0101 and 721FRET.A0101, respectively) were isolated by FACS to recover strong FRET expressers and HLA surface expression (based on staining with APC-conjugated anti-human MHC, clone G46-2.6, BD Bioscience).

### Creation of single-minigene/minigene library-expressing sAPC lines

Minigene-expressing target cells were produced by transducing pre-purified KFRET.A0101 or 721FRET.A0101 with viral vector produced using pMinigene-IRES-mStrawberry format constructs. For single-minigene controls (i.e. MAGEA3 and TTN minigene-expressing lines), KFRET.A0101 were transduced at an MOI of 0.3 KIU/cell and 721FRET.A0101 was transduced at an MOI of 1 KIU/cell under the same scale and conditions as HLA-FRET cell line creations. For WPC minigene libraries in KFRET.A0101, cells were transduced with library vector at MOI = 0.2 over 3.5 × 10^8^ starting cells (in G-Rex format) for low-MOI approach or MOI = 7 over 1.0 × 10^7^ starting cells for high-MOI approaches. For 721FRET.A0101 library transduction, 8 × 10^6^ cells were infected with 20 KIU/cell in serum-free OptiMEM (to prevent cluster formation during viral infection). After 24 hours, transduced 721FRET.A0101 were centrifuged and re-seeded in complete medium for continued culture. For 1^st^ round WES minigene libraries in KFRET.A0101, cells were transduced with library vector at MOI = 3 over 8 × 10^6^ starting cells and, in 2^nd^ round panning libraries, at MOI = 0.7 over 1.2 × 10^7^ starting cells. For all minigene-expressing cell lines, purity sorting was conducted by selecting RFP^+^ cells. Minigene library-expressing cell lines were purity-sorted 5–7 days after transduction (yielding a minimum of 1.5 × 10^7^ founder clones for WPC format; 2.5 × 10^7^ for WES/panning format) and cryopreserved 5–7 days after purity sorting to minimize library drift (stored at 2.5 × 10^7^ cells/vial to mitigate bottlenecking).

### Co-culture setup for Tope-seq experiments

For large-scale library screening Tope-seq experiments, TCR-T cells were always freshly prepared starting 11–14 days prior from primary human PBMC, while library-expressing sAPC were always thawed 5 days prior to assay setup using an inoculum of >2.5 × 10^7^ cells in a 300 mL media volume. Replicate experiments were always performed on different days, using TCR-T cells produced from different donor sources, and library target cell lines prepared from separate transductions. To prepare experimental co-cultures, target and effector cells were adjusted to a density of 2 × 10^6^ cells/mL and 3 × 10^6^ cells/mL, respectively in fresh, pre-warmed media (TCR-T were adjusted to a higher density to account for an average 65% TCR transduction rate). For KFRET based library cell lines, 50 mL of each were combined and gently swirled to mix. Cell mixture was distributed uniformly to all wells of 10 x 96-well U-bottom plates and incubated for 12 hours. For library screens involving 721FRET based targets, where very short (<1hr) co-culture periods were needed, plates were prepared in staggered batches: target and effector cells were each adjusted to a density of 2 × 10^6^ cells/mL or 3 × 10^6^ cells/mL, respectively, in fresh, pre-warmed media and 15 mL of each were combined in a 50 mL centrifuge tube. Cell mixture was gently inverted to mix and distributed uniformly to all wells of 3 × 96-well U-bottom plates. During sorting of first batch, the next batch was assembled, incubated, and sample prepped. Once complete, new batch was immediately loaded for sorting. For each replicate, three staggered batches were prepared and run. In all screening experiments, a plate of individual control co-cultures was assembled, consisting of TCR-T cells against single-minigene and no-minigene controls. This was done by adjusting each target and effector population to a density of 2 × 10^6^ cells/mL in fresh, pre-warmed media, combining 100 μL of each test pair to run, and plating in 2 wells of a 96-well U-bottom plate per control condition to be included. For each control condition, a pair of wells was left unmixed and combined immediately before flow cytometry to serve as a T_0_ loading control. On conclusion of co-culture period, cells were stained on-plate with 0.5% BV785-conjugated anti-CD8 antibody, clone SK1 (Biolegend) and 0.1% Fixable Viability Dye eFluor 780 (ThermoFisher) for 15 minutes at 4°C before transferring co-culture wells to tubes, washing cells 1X in 10 volumes cold PBS, and resuspending in cold PBS + 2% FBS. Prepared co-culture samples were kept on ice and taken immediately for flow cytometry/FACS.

### Flow cytometry/FACS

All FACS was performed BD FACSAria Fusion running BD FACSDiva v9.0 software. Gating was performed by monitoring FVD eFluor780 channel (ex. 640, em. 780/60), RFP channel (ex. 561, em 610/20 + 600LP), YFP channel (ex. 488, em. 530/30 + 505LP), PerCP-Cy5.5 channel (ex. 488/em. 695/40 + 655LP), BV785 channel (ex. 405, em. 780/60 + 750LP), FRET channel (ex. 405, em. 525/50 + 505LP), and/or CFP channel (ex. 405, em. 450/50) according to experiment design. Cytometric analyses were performed on BD LSRFortessa and quantitated using BD FlowJo v10.

### Minigene sequencing

Cells sorted from matched FRET-shifted and unshifted gates in each replicate Tope-seq screening experiment were immediately pelleted and lysed using DNAzol reagent. Ethanol precipitation, washing, and re-solubilization was performed according to manufacturer’s protocol. To prepare samples for sequencing, two rounds of PCR were conducted. In the first, 50% of all recovered gDNA per sample was input as template across multiple parallel reactions (capping the amount of template to be added per 100 µL reaction at the lower of 10 µL or 250 ng). First round PCR was run for 25 cycles with Phusion polymerase (NEB) and Illumina adapter primers, F: 5’-ACGACGCTCTTCCGATCT-3’ and R: 5’-CGTGTGCTCTTCCGATCT-3’. For second round PCR, 100 ng of column-cleaned first round product was used as template and amplified for 5 cycles using primers from NEBNext Multiplex Oligo Set, uniquely barcoding each gate sample of each replicate experiment. Prepared libraries were subject to PE150 sequencing on the Illumina HiSeq 4000 platform at off-site service provider, Genewiz.

### Data analysis

For WPC library-based screening, matched Shifted and Unshifted gate minigene sequencing reads were trimmed with Cutadapt v1.18 (default settings), paired-end merged with FLASh v1.2.11 (--min-overlap 20 --max-overlap 150 --max-mismatch-density 0.2 --allow-outies), quality filtered with FASTX-toolkit (keeping reads with 90% of bases > Q20), and error corrected/mapped to synthesized minigenes using Starcode v1.1 (-d 4 in spheres mode for collapsing errors; -d 3 in spheres mode for aligning to canonical minigene designs). Motif-building analyses were conducted using NetMHC4.0 (default parameters) and GibbsCluster2.0 (lambda 0, sigma 0, trash cluster threshold 10). For WES libraries, Illumina adapters were removed using Trim Galore v.0.6.6 in paired-end mode. A quality score of 20 was used for trimming low quality bases from the rear ends. Reads were aligned to the hg19 human reference genome using BWA-MEM2 v.2.2.1. Reads were then processed using samtools v.1.9 by name ordering and collating, fixing mate information, position ordering, marking duplicates and indexing. Processed reads were counted by using a sliding reference window to count all in-frame minigene fragments traversing each peptide-coding window of defined size in the entire coding genome. Data handling and visualization for all sequencing experiments was performed with R v4.4.1, seqinr v4.2-36, and ggplot2 v3.5.1.

## Data Availability

Raw and processed flow cytometry data generated in this study are available from corresponding authors on reasonable request. Raw .fastq files from Tope-seq experiments are available for download from the NCBI: https://www.ncbi.nlm.nih.gov/bioproject/PRJNA1258577.

## References

[1] Schreiber RD Old LJ Smyth MJ . Cancer immunoediting: integrating immunity’s roles in cancer suppression and promotion. Science. (2011) 331:1565-70. doi: 10.1126/science.1203486, PMID: 21436444

[2] Klein L Petrozziello E . Antigen presentation for central tolerance induction. Nat Rev Immunol. (2025) 25:57–72. doi: 10.1038/s41577-024-01076-8, PMID: 39294277

[3] Tsimberidou A-M Van Morris K Vo HH Eck S Lin Y-F Rivas JM . T-cell receptor-based therapy: an innovative therapeutic approach for solid tumors. J Hematol Oncol.J Hematol Oncol. (2021) 14:1–22. doi: 10.1186/s13045-021-01115-0, PMID: 34193217 PMC8243554

[4] Klebanoff CA Chandran SS Baker BM Quezada SA Ribas A . T cell receptor therapeutics: immunological targeting of the intracellular cancer proteome. Nat Rev Drug Discov. (2023) 22:996–1017. doi: 10.1038/s41573-023-00809-z, PMID: 37891435 PMC10947610

[5] Linnemann C van Buuren MM Bies L Verdegaal EME Schotte R Calis JJA . High-throughput epitope discovery reveals frequent recognition of neo-antigens by CD4+ T cells in human melanoma. Nat Med. (2015) 21:81–5. doi: 10.1038/nm.3773, PMID: 25531942

[6] Wooldridge L Ekeruche-Makinde J van den Berg HA Skowera A Miles JJ Tan MP . A single autoimmune T cell receptor recognizes more than a million different peptides. J Biol Chem. (2012) 287:1168–77. doi: 10.1074/jbc.M111.289488, PMID: 22102287 PMC3256900

[7] Bridgeman JS Sewell AK Miles JJ Price DA Cole DK . Structural and biophysical determinants of αβ T-cell antigen recognition. Immunology. (2012) 135:9–18. doi: 10.1111/j.1365-2567.2011.03515.x, PMID: 22044041 PMC3246648

[8] Das DK Feng Y Mallis RJ Li X Keskin DB Hussey RE . Force-dependent transition in the T-cell receptor β-subunit allosterically regulates peptide discrimination and pMHC bond lifetime. Proc Natl Acad Sci USA. (2015) 112:1517–22. doi: 10.1073/pnas.1424829112, PMID: 25605925 PMC4321250

[9] James JR Vale RD . Biophysical mechanism of T-cell receptor triggering in a reconstituted system. Nature. (2012) 487:64–9. doi: 10.1038/nature11220, PMID: 22763440 PMC3393772

[10] Bentzen AK Marquard AM Lyngaa R Saini SK Ramskov S Donia M . Large-scale detection of antigen-specific T cells using peptide-MHC-I multimers labeled with DNA barcodes. Nat Biotechnol. (2016) 34:1037–45. doi: 10.1038/nbt.3662, PMID: 27571370

[11] Gee MH Han A Lofgren SM Beausang JF Mendoza JL Birnbaum ME . Antigen identification for orphan T cell receptors expressed on tumor-infiltrating lymphocytes. Cell. (2018) 172:549–63. doi: 10.1016/j.cell.2017.11.043, PMID: 29275860 PMC5786495

[12] Martinez RJ Andargachew R Martinez HA Evavold BD . Low-affinity CD4+ T cells are major responders in the primary immune response. Nat Commun. (2016) 7:13848. doi: 10.1038/ncomms13848, PMID: 27976744 PMC5234832

[13] Rius C Attaf M Tungatt K Bianchi V Legut M Bovay A . Peptide–MHC class I tetramers can fail to detect relevant functional T cell clonotypes and underestimate antigen-reactive T cell populations. J Immunol. (2018) 200:2263–79. doi: 10.4049/jimmunol.1700242, PMID: 29483360 PMC5857646

[14] Nielsen M Eugster A Jensen MF Goel M Tiffeau-Mayer A Pelissier A . Lessons learned from the IMMREP23 TCR-epitope prediction challenge. ImmunoInformatics. (2024) 16:100045. doi: 10.1016/j.immuno.2024.100045

[15] Spear TT Evavold BD Baker BM Nishimura MI . Understanding TCR affinity, antigen specificity, and cross-reactivity to improve TCR gene-modified T cells for cancer immunotherapy. Cancer Immunol Immunother. (2019) 68:1881–9. doi: 10.1007/s00262-019-02401-0, PMID: 31595324 PMC11028285

[16] Sharma G Rive CM Holt RA . Rapid selection and identification of functional CD8^+^ T cell epitopes from large peptide-coding libraries. Nat Commun. (2019) 10. doi: 10.1038/s41467-019-12444-7, PMID: 31591401 PMC6779888

[17] Sharma G Round J Teng F Ali Z May C Yung E . A synthetic cytotoxic T cell platform for rapidly prototyping TCR function. NPJ Precis Oncol. (2024) 8:182. doi: 10.1038/s41698-024-00669-9, PMID: 39160299 PMC11333705

[18] Cameron BJ Gerry AB Dukes J Harper JV Kannan V Bianchi FC . Identification of a Titin-derived HLA-A1-presented peptide as a cross-reactive target for engineered MAGE A3-directed T cells. Sci Transl Med. (2013) 5:1–11. doi: 10.1126/scitranslmed.3006034, PMID: 23926201 PMC6002776

[19] Karanikas V Lurquin C Colau D van Baren N De Smet C Lethé B . Monoclonal anti-MAGE-3 CTL responses in melanoma patients displaying tumor regression after vaccination with a recombinant canarypox virus. J Immunol. (2003) 171:4898–904. doi: 10.4049/jimmunol.171.9.4898, PMID: 14568971

[20] Shimizu Y Geraghty DE Koller BH Orr HT DeMars R . Transfer and expression of three cloned human non-HLA-A,B,C class I major histocompatibility complex genes in mutant lymphoblastoid cells. Proc Natl Acad Sci USA. (1988) 85:227–31. doi: 10.1073/pnas.85.1.227, PMID: 3257565 PMC279517

[21] Jurtz V Paul S Andreatta M Marcatili P Peters B Nielsen M . NetMHCpan-4.0: improved peptide–MHC class I interaction predictions integrating eluted ligand and peptide binding affinity data. J Immunol. (2017) 199:3360–8. doi: 10.4049/jimmunol.1700893, PMID: 28978689 PMC5679736

[22] Andreatta M Lund O Nielsen M . Simultaneous alignment and clustering of peptide data using a Gibbs sampling approach. Bioinformatics. (2013) 29:8–14. doi: 10.1093/bioinformatics/bts621, PMID: 23097419

[23] Vazquez-Lombardi R Jung JS Schlatter FS Mei A Rodrigues Mantuano N Bieberich F . High-throughput T cell receptor engineering by functional screening identifies candidates with enhanced potency and specificity. Immunity. (2022) 55:1–14. doi: 10.1016/j.immuni.2022.09.004, PMID: 36174557

[24] Uhlén M Fagerberg L Hallström BM Lindskog C Oksvold P Mardinoglu A . Tissue-based map of the human proteome. Science. (2015) 347:1260419–1260419. doi: 10.1126/science.1260419, PMID: 25613900

[25] Zhuang X Woods J Ji Y Scheich S Mo F Rajagopalan S . Functional genomics identifies N-acetyllactosamine extension of complex N-glycans as a mechanism to evade lysis by natural killer cells. Cell Rep. (2024) 43:114105. doi: 10.1016/j.celrep.2024.114105, PMID: 38619967 PMC11170631

[26] Wu CH Liu IJ Lu RM Wu HC . Advancement and applications of peptide phage display technology in biomedical science. J Biomed Sci. (2016) 23:1–14. doi: 10.1186/s12929-016-0223-x, PMID: 26786672 PMC4717660

[27] Linette GP Stadtmauer EA Maus MV Rapoport AP Levine BL Emery L . Cardiovascular toxicity and titin cross-reactivity of affinity-enhanced T cells in myeloma and melanoma. Blood. (2013) 122:863–71. doi: 10.1182/blood-2013-03-490565, PMID: 23770775 PMC3743463

[28] Coles CH Mulvaney RM Malla S Walker A Smith KJ Lloyd A . TCRs with distinct specificity profiles use different binding modes to engage an identical peptide–HLA complex. J Immunol. (2020) 204:1943–53. doi: 10.4049/jimmunol.1900915, PMID: 32102902 PMC7086387

[29] Sanderson JP Crowley DJ Wiedermann GE Quinn LL Crossland KL Tunbridge HM . Preclinical evaluation of an affinity-enhanced MAGE-A4-specific T-cell receptor for adoptive T-cell therapy. OncoImmunology. (2019) 9:1682381. doi: 10.1080/2162402X.2019.1682381, PMID: 32002290 PMC6959444

[30] Wooldridge L Laugel B Ekeruche J Clement M van den Berg HA Price DA . CD8 controls T cell cross-reactivity. J Immunol. (2010) 185:4625–32. doi: 10.4049/jimmunol.1001480, PMID: 20844204 PMC3018649

[31] Riley TP Hellman LM Gee MH Mendoza JL Alonso JA Foley KC . T cell receptor cross-reactivity expanded by dramatic peptide–MHC adaptability. Nat Chem Biol. (2018) 14:934–42. doi: 10.1038/s41589-018-0130-4, PMID: 30224695 PMC6371774

[32] Kina E Larouche J-D Thibault P Perreault C . The cryptic immunopeptidome in health and disease. Spec Issue Microproteins. (2025) 41:162–9. doi: 10.1016/j.tig.2024.09.003, PMID: 39389870

[33] Sharma G . Novel *in vitro* methods for the discovery of functional T-cell receptor epitopes from large peptide-coding libraries (T). Vancouver, BC Canada: University of British Columbia (2018). doi: 10.14288/1.0375763

