# Supplementary material for: Comprehensive self-antigen screening to assess cross-reactivity in promiscuous T-cell receptors
Source: Front Immunol. 2026 Jan 5;16:1719827. doi: 10.3389/fimmu.2025.1719827 (PMC12813212; doi:10.3389/fimmu.2025.1719827)
Supplement: Supplementary file 2 [file SupplementaryFile1.pdf]

**Supplementary Table 1. Summary of WPC plasmid library quality assessment by NGS.** A sample of WPC minigene library plasmid was input for Illumina library preparation and sequencing to quantify non-mappable reads and design coverage.	
	**Count**
Quality filtered reads	169,864,738
Mapped to reference	165,969,580 (97.7% of total quality filtered reads)
Reference coverage	522,045 (97.4% of designed reference library)

Supplementary Table 2. Summary of low-MOI KFRET.A0101.WPC quality assessment by NGS. An initial low MOI (MOI=0.2) strategy was used to favor probabilistically the production of no more than a single minigene integration per cell. Samples from each of four different replicate transductions, consisting of 2-3 x 10^7 cells, were lysed and subject to minigene recovery by PCR from isolated gDNA prior to Illumina sequencing. For each individual replicate, coverage of designed library and average read depth for each minigene was quantitated. Reads across all four replicates were also merged and used to quantify the design library coverage as a union dataset. On average, replicate samples were found to contain 86.9% of designed minigene sequences. When considering the union of all four replicates, the increased depth from combining the data resulted in library coverage roughly similar to the source plasmid. We reasoned, therefore, that bottlenecking was not introduced at the transduction step since sampling more cells resulted in near complete coverage.

	**Replicate 1**	**Replicate 2**	**Replicate 3**	**Replicate 4**	**Union**
Quality filtered, mapped reads	247,448,584	193,723,485	126,096,987	125,836,195	
Reference coverage (count)	416,474	473,256	486,634	487,257	518,993
Reference coverage (%)	77.7	88.3	90.8	90.9	96.9
Average read depth per minigene	594.1	409.3	259.1	258.3	

Supplementary Table 3. Summary of high-MOI KFRET.A0101.WPC quality assessment by NGS. A sample of WPC library-expressing target cells generated by high MOI strategy were taken from each of two different replicate transductions. For each sample, 2-3 x 10^7 cells were lysed and subject to minigene recovery by PCR from isolated gDNA. For each individual replicate, coverage of designed library and average read depth for each minigene was quantitated.

	**Replicate 1**	**Replicate 2**
Quality filtered, mapped reads	176,219,645	178,408,919
Reference coverage (count)	524,078	522,862
Reference coverage (%)	97.8	97.6
Average read depth per minigene	336.2	341.2

**Supplementary Table 4. Summary of KFRET.A0101.WES quality assessment by NGS.** A sample of 2 x 10^7 cells were lysed and subject to minigene recovery by PCR from isolated gDNA. Raw reads were quality filtered and mapped to hg19 human reference genome. The WES library was estimated to encode 100X coverage of the human exome (based on number of unique mapped reads detected, average fragment length, and an estimated exome size of 50 Mbp). The average read depth of individual minigenes was also quantified and found to be much lower than that typically observed in WPC libraries, indicating that epitope redundancy within libraries of WES format is derived primarily from high unique minigene fragment count.	
Raw reads	114,717,984
Quality filtered, mapped reads	108,698,439 (94.8% of total reads)
Average fragment size (bp)	212
Num. unique fragments	25,205,103
Estimated exome coverage	>100X
Average read depth per minigene	4.3

Supplementary Table 5. Quantification of WES library bottlenecking in 2nd round libraries. The number of unique minigenes detected in 2nd round screening of a3a and EB81.103 TCR-T cells after re-cloning Shifted gate minigenes from initial 1st round screens. In both cases, a substantial fraction of minigenes from the original source WES minigene library are not re-detected in 2nd round experiments.

	**a3a-screened 2nd round library**	**EB81.103-screened 2nd round library**
Quality filtered, mapped reads	63,386,248	185,812,985
Num. unique fragments	1,043,412	1,935,310
Percentage of original library	4.1%	7.7%

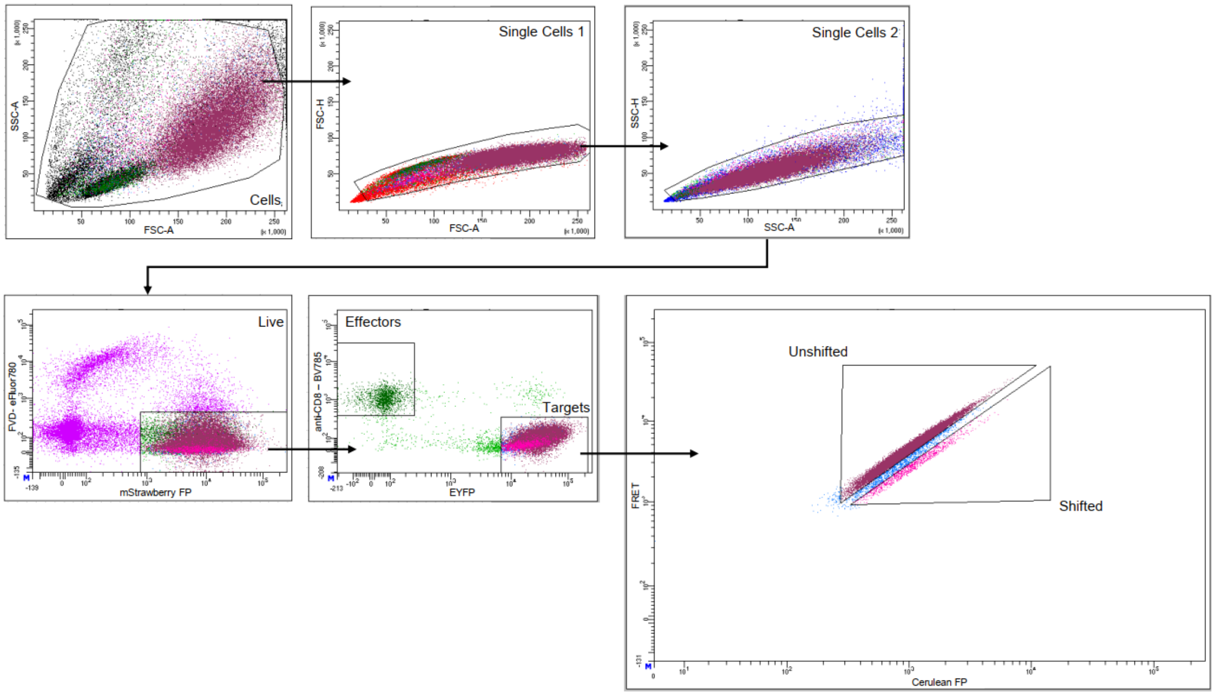

Supplementary Figure 1. Representative gating strategy for FRET-shift flow cytometry in Tope-seq experiments. A common sorting template was used to perform two-way FACS for capturing matched Shifted and Unshifted gate cells in all library screens used to generate Tope-seq data. Intact, single cells are first selected using sequential forward- and side-scatter gates. Viable cells are then selected by gating on cells negative for FVD780 vital staining dye and positive for mStrawberry RFP living color. Effector and target cells are separated by CD8-BV785 staining and EYFP expression. Unshifted and Shifted gates are drawn by adjusting gate boundaries to keep background FRET-shift in T_0 or targets-only control samples to <1%.

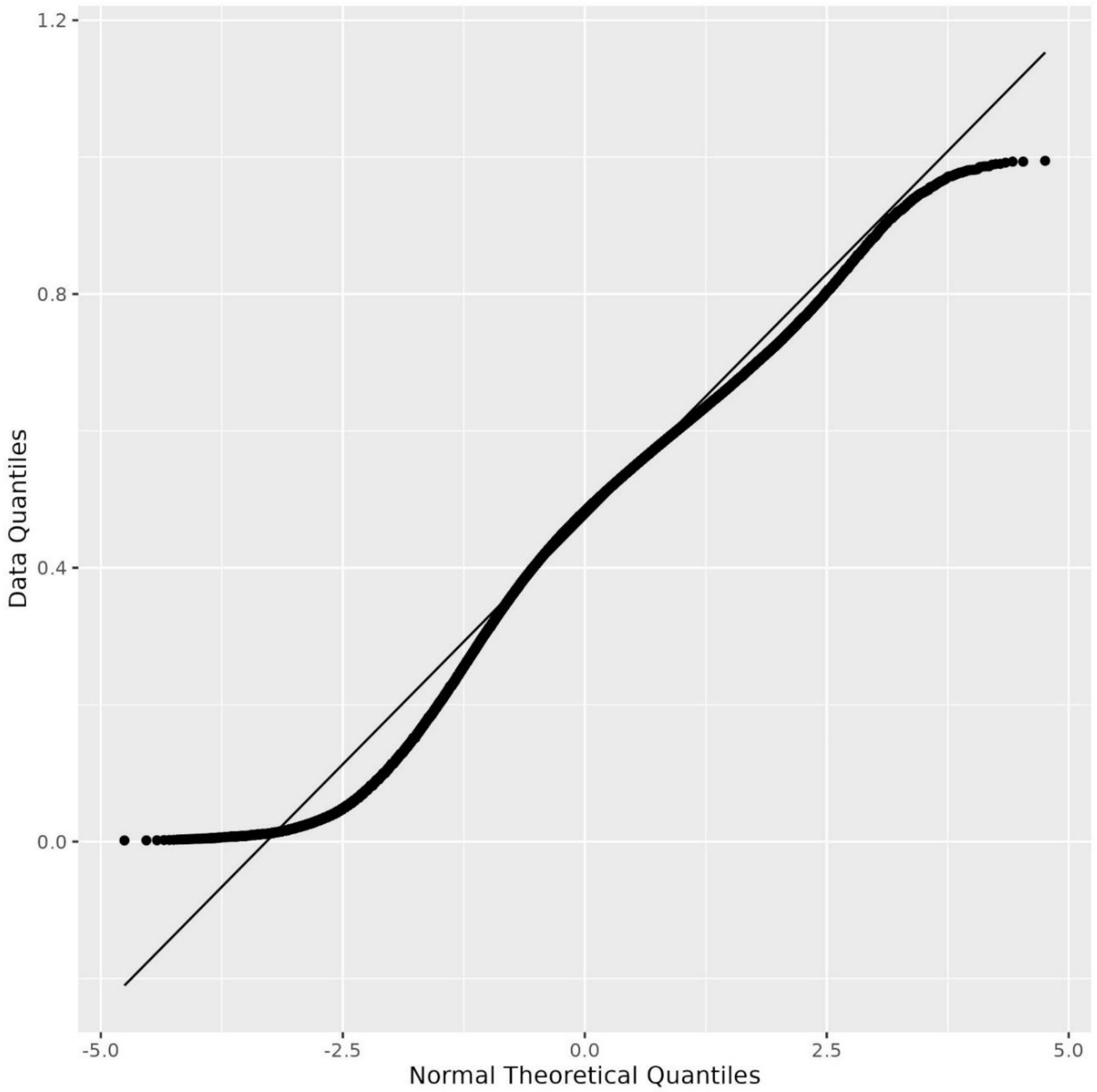

Supplementary Figure 2. Quantile-quantile plot to estimate normality of Tope-seq scores. To confirm if Z-score calculation was suitable for significance, normality testing was done on enrichment scores calculated from relative frequency of normalized read count for each minigene/peptide sequence in Shifted gate as a proportion of its normalized read counts in Shifted + Unshifted. Strong linear correlation in a representative example, shown here, between theoretical quantiles and experimental quantiles empirically confirmed that the relative frequency scoring statistic used in this study are approximately normally distributed.

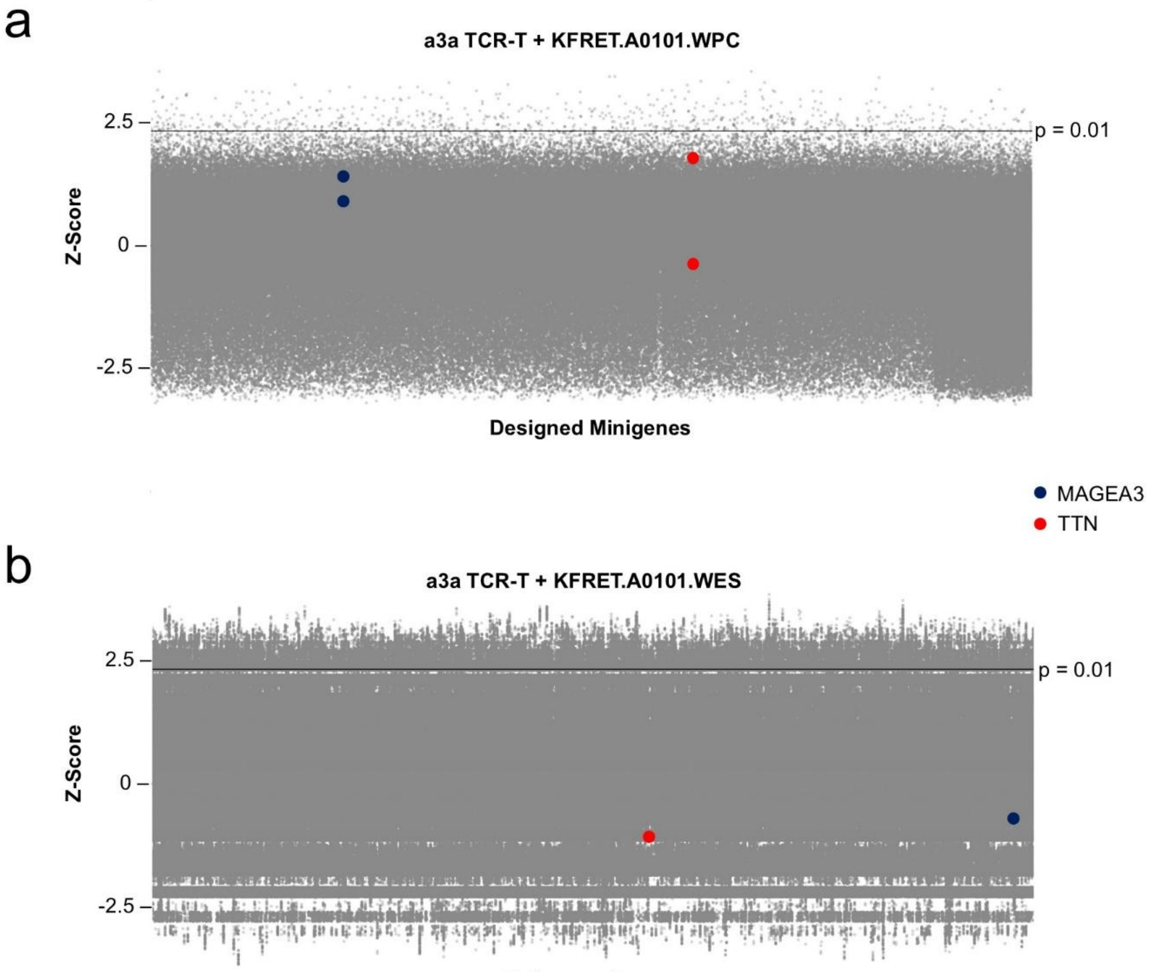

Supplementary Figure 3. Tope-seq assessment of a3a TCR-T using unedited K562 target chassis. Tope-seq readout from initial test of a3a TCR-T cells co-cultured with KFRET.A0101 cells containing either the WPC library (a) or WES library (b). In both experiments, an effector/target ratio of 1:1 was used to assemble co-cultures. For each screen, >6 x 10^7 target library cells were incubated with TCR-T cells for a period of 12 hours prior to FACS isolation of Shifted and Unshifted cells. Recovered cells were subject to gDNA purification and minigene amplification before Illumina sequencing. The frequency of reads in Shifted relative the normalized read counts in Shifted + Unshifted for each minigene (for (a)) or 9-mer coding genomic locus (for (b)) were computed and used to calculate Z-scores plotted on y-axes. Each grey point represents an individual minigene or 9-mer peptide coding locus. The control MAGEA3 and TTN epitope-containing minigenes (for a) or minimal epitopes from MAGEA3 and TTN (for b) are highlighted according to the figure legend. Significance threshold representing $p = 0.01$ is indicated on y-axes as a solid line (Z-scores above line are $p < 0.01$).

a

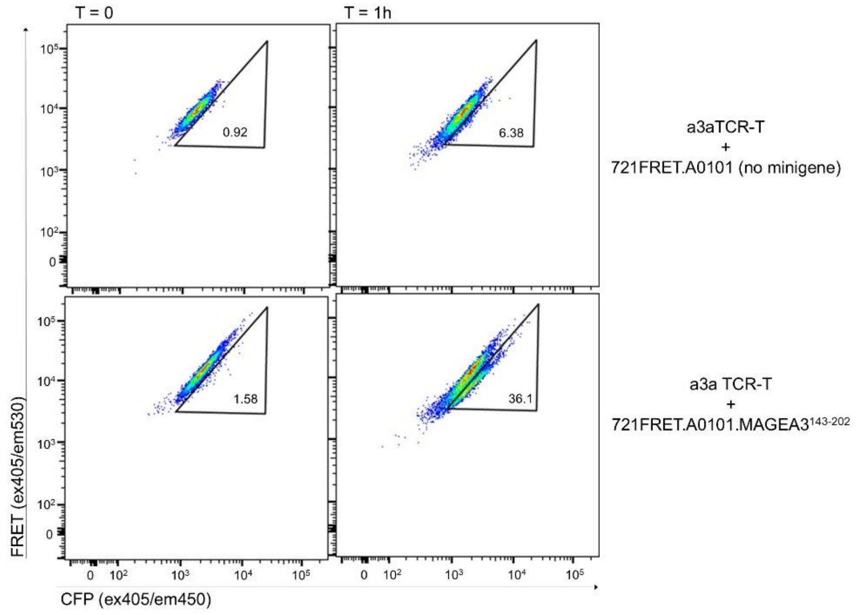

b

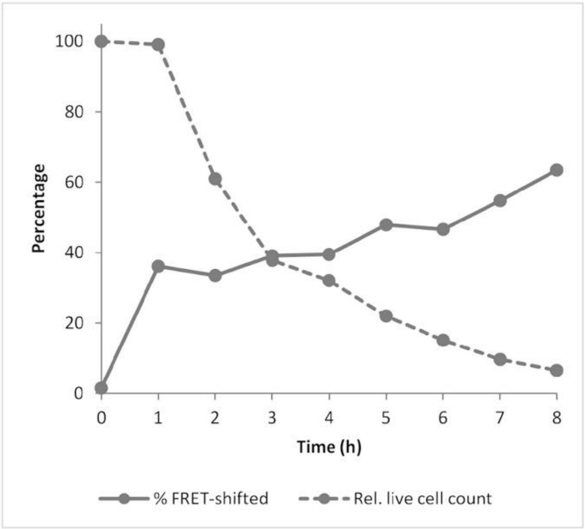

Supplementary Figure 4. Assessment of a3a TCR-T + 721FRET.A0101.MAGEA3$^{143\text{-}202}$ co-cultures. (a) Engineered 721.221 cells expressing HLA-A*01:01/GZMB FRET reporter transgenes with or without a positive control MAGEA3$^{143\text{-}202}$ minigene cassette were prepared and co-cultured with primary cytotoxic T cells freshly isolated by FACS purification CD8+ CD4- CD56- T cells from healthy donor PBMC, activated by plate-bound anti-CD3/28 stimulation, and transduced with a3a TCR lentivirus. Co-cultures were conducted at a 1.5:1 ratio (to compensate for 68% TCR transduction efficiency) for 1 hour and analyzed by flow cytometry to quantify GZMB-

induced FRET-shifting in targets. (b) To further validate 721.221 cell line for use as a target sAPC to use in Tope-seq experiments, a time course experiment was performed to determine the safe-sorting window within which GZMB-loaded target cells should be collected before being lost to apoptosis cell death and degradation. Reactions were assembled by plating 2×10^5 of each cell line into 2 separate wells of a U-bottom 96-well plate. An effector/target well pair was prepared for each of the 8 time points investigated and left unmixed until co-culture periods were initiated at the appropriate time prior to flow cytometry by combining effector and target wells and re-distributing co-cultures to both wells. Flow cytometry was conducted on all samples in a single batch at experiment conclusion. Percent FRET-shift signal and percent survival (live target cell counts normalized to matched T_0 control) are displayed.
